# The Distribution of Several Genomic Virulence Determinants Does Not Corroborate the Established Serotyping Classification of *Bacillus thuringiensis*

**DOI:** 10.3390/ijms22052244

**Published:** 2021-02-24

**Authors:** Anton E. Shikov, Yury V. Malovichko, Arseniy A. Lobov, Maria E. Belousova, Anton A. Nizhnikov, Kirill S. Antonets

**Affiliations:** 1 Laboratory for Proteomics of Supra-Organismal Systems, All-Russia Research Institute for Agricultural Microbiology (ARRIAM), 196608 St. Petersburg, Russia; a.shikov@arriam.ru (A.E.S.); yu.malovichko@arriam.ru (Y.V.M.); m.belousova@arriam.ru (M.E.B.); a.nizhnikov@arriam.ru (A.A.N.); 2 Faculty of Biology, St. Petersburg State University (SPbSU), 199034 St. Petersburg, Russia; arseniylobov@gmail.com; 3 Laboratory of Regenerative Biomedicine, Institute of Cytology of the Russian Academy of Science, 194064 St. Petersburg, Russia

**Keywords:** *Bacillus thuringiensis*, *Bt*, virulence factors, proteomics, 2D-DIGE, mass spectrometry, phylogeny, pangenome, phylogenomics, serotyping, flagellin

## Abstract

*Bacillus thuringiensis*, commonly referred to as *Bt*, is an object of the lasting interest of microbiologists due to its highly effective insecticidal properties, which make *Bt* a prominent source of biologicals. To categorize the exuberance of *Bt* strains discovered, serotyping assays are utilized in which flagellin serves as a primary seroreactive molecule. Despite its convenience, this approach is not indicative of *Bt* strains’ phenotypes, neither it reflects actual phylogenetic relationships within the species. In this respect, comparative genomic and proteomic techniques appear more informative, but their use in *Bt* strain classification remains limited. In the present work, we used a bottom-up proteomic approach based on fluorescent two-dimensional difference gel electrophoresis (2D-DIGE) coupled with liquid chromatography/tandem mass spectrometry(LC-MS/MS) protein identification to assess which stage of *Bt* culture, vegetative or spore, would be more informative for strain characterization. To this end, the proteomic differences for the *israelensis*-attributed strains were assessed to compare sporulating cultures of the virulent derivative to the avirulent one as well as to the vegetative stage virulent bacteria. Using the same approach, virulent spores of the *israelensis* strain were also compared to the spores of strains belonging to two other major *Bt* serovars, namely *darmstadiensis* and *thuringiensis*. The identified proteins were analyzed regarding the presence of the respective genes in the 104 *Bt* genome assemblies available at open access with serovar attributions specified. Of 21 proteins identified, 15 were found to be encoded in all the present assemblies at 67% identity threshold, including several virulence factors. Notable, individual phylogenies of these core genes conferred neither the serotyping nor the flagellin-based phylogeny but corroborated the reconstruction based on phylogenomics approaches in terms of tree topology similarity. In its turn, the distribution of accessory protein genes was not confined to the existing serovars. The obtained results indicate that neither gene presence nor the core gene sequence may serve as distinctive bases for the serovar attribution, undermining the notion that the serotyping system reflects strains’ phenotypic or genetic similarity. We also provide a set of loci, which fit in with the phylogenomics data plausibly and thus may serve for draft phylogeny estimation of the novel strains.

## 1. Introduction

*Bacillus thuringiensis* (*Bt*) is a soil-dwelling spore-forming bacterium belonging to the so-called *Bacillus cereus sensu lato* group of *Bacillus* species. Unlike its closest relatives, *Bt* is mostly known as a pathogen of numerous insect species and other invertebrate hosts. Due to its specificity and pesticidal properties, *Bt* has been widely used as a source for biologicals production since the 1920s [1]. Extensive use of *Bt*-derived pesticides resulted in the isolation of numerous strains different in their phenotypes and host range. In order to categorize them, several approaches have been proposed [2,3], of which a serotyping-based classification remains a predominant one [4,5]. By definition, this approach implies cell agglutination induction by antibodies recognizing variable epitopes of flagellin, a structural protein of flagellar filament, with strains aggregated into serovars depending on their shared affinity to the specific antibody type. By the end of the 20th century, approximately 50,000 *Bt* strains had been isolated and contained worldwide [6], comprising representatives of 80 different serovars [5]. By 2017, the number of serovars had increased up to 86 [7].

Notwithstanding its widespread use, the serotyping classification was frequently claimed to be irreflective of the strains’ genetic, phenotypic, or evolutionary commonality [8]. In this regard, the use of comparative genomic and/or proteomic techniques provides a more reliable way of grouping *Bt* strains for practical purposes. To a certain extent, the proteomic approach was incorporated in the classical *Bt* strain nomenclature since the shape of crystaloferous inclusions is used as a diagnostic feature [4]. The use of crystal morphology may be further reinforced by molecular analysis of the endotoxin genes repertoire as well as by the involvement of numerous other virulence genes contributing to strains’ pathogenicity [9]. Although most of these determinants are detectable at the genomic level, proteomic assays may provide essential evidence for their distribution. To date, both genomic and proteomic techniques are utilized in *Bt* studies, although the number of comparative proteomics papers [10,11] is considerably smaller than that of works on comparative genomics [12,13,14,15]. Ideally, the genomic and proteomic approaches should be used in parallel in order to detect virulence factors that are either missing in the proteome screening [16] or misannotated in the genomic data [17]. This combined approach is especially relevant when concerning the identification of Cry toxins, which tend to fall out of the scope in both proteomic [11,16] and genomic [18] studies. However, the phylogenetic outreach of virulence profiles should be interpreted with caution, provided that the high rate of horizontal gene transfer among the *Bc* group members often leads to an intermingling of virulent phenotypes both between *Bt* strains [19,20] and *Bt* and other *Bacillus* species [21,22].

Although proteomics assays have been carried out on *Bt* to define the toxicity agents of particular strains, none of them addresses the correlation of particular proteins with the serovar attribution. In this work, we pried whether proteins detectable using common proteomics techniques in spores or vegetative cells can be used as discriminating markers for serological groups of *Bt*. To this end, we analyzed the proteomes of three *Bt* strains belonging to different serovars as well as the proteome of one non-virulent descendant of serovar *israelensis* using fluorescent two-dimensional difference protein gel electrophoresis (2D-DIGE, [23]) with “bottom-up” protein identification by HPLC coupled with tandem mass spectrometry [24,25]. When analyzing 2D-DIGE gels, we primarily focused on differentially produced major proteins to test the applicability and efficacy of the “bottom-up” proteomics approach for discerning between *Bt* serotype groups. The identification of such major protein markers could provide insights into easy serotype delineation free from limitations of the agglutination-base method. For the proteins annotated, the respective gene identifiers were used to detect gene presence across the *Bt* pangenome. Further check of core genes’ individual phylogeny was undertaken in order to elucidate whether these genes reflect strain phylogeny and serological classification.

## 2. Results

### 2.1. Virulence Factors Are Enriched in the Proteomes of Bt var. israelensis Virulent Sporulating Cultures Compared to the Avirulent and Vegetative Ones

**Figure 1:**
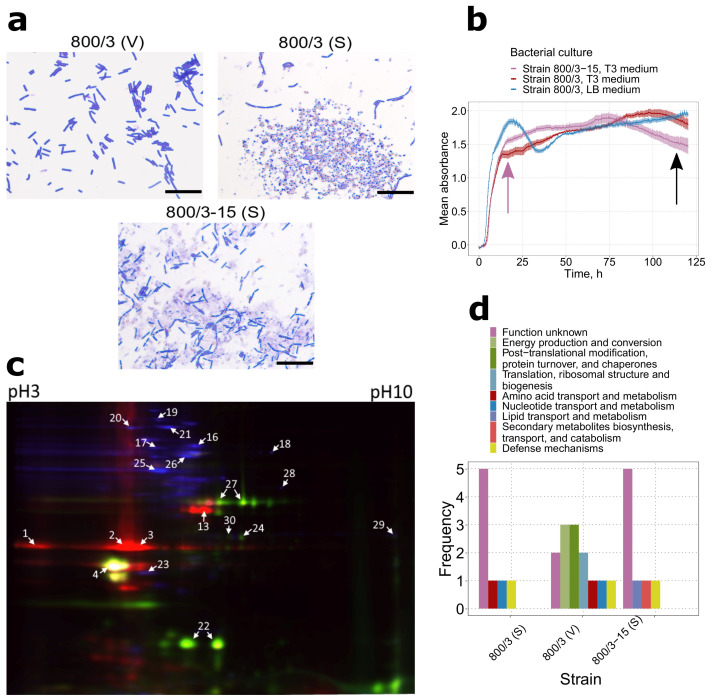
Proteomic signatures associated with different stages of the lifecycle in two strains belonging to serovar *israelensis*. (**a**) Microscope images of strain 800/3 vegetative culture (V), strain 800/3 virulent sporulating (S) culture, and strain 800/3-15 avirulent sporulating (A) culture All photos were taken at ×1000 magnitude in transmitted light. Scale bars are given as black rectangles and denote 20 μm. Parasporal inclusions in the strain 800/3 spores were stained with Coomassie Blue. (**b**) Growth curves of strain 800/3 and 800/3-15 cultures grown on T3 medium and strain 800/3 vegetative cultures grown on LB solution medium. The purple arrow marks the time of the vegetative culture’s protein extraction, the black arrow—spore cultures (**c**) 2D-DIGE image corresponding to the overlapping Cy2, Cy3, and Cy5 fluorochrome channels of serovar *israelensis* proteomes. Red light channel indicates– proteins from strain 800/3, blue—strain 800/3 vegetative cells proteins, and green—strain 800/3-15 spore proteins. (**d**) The COG term distribution among the proteins detected with ESI-MS. COG annotation was assigned to the reference sequences by sequence homology using eggNOG mapper.

Being large and widely used for dipteran pest control, serovar *israelensis* was selected to assess the differences in the proteomic profiles between the vegetative cells and sporulating culture. Apart from the virulent strain 800/3 previously reported to harbor *cry4* and *cry11* genes and active against the insects of Diptera order [26,27], spores of an avirulent descendant strain, 800/3-15, were analyzed in order to assess the differences in the virulence factors production (Figure 1a). To ensure that the cultures were sampled at the proper time points, absorption profiles of the growing cultures were analyzed (Figure 1b). The curvature of the graph corresponded with the time the culture was sampled in all three cases, which stood for the stationary condition in the vegetative culture and cessation of sporulation in the sporulating cultures.

The proteomes of all three samples were visualized with the 2D-DIGE technique (Figure 1c). Further mass spectrometry of 32 most prominent protein spots and manual analysis of the obtained results revealed a total of 19 non-redundant proteins detected (Table S1). Of these, 13 entries were found in the proteome of strain 800/3 vegetative culture, while eight and seven proteins were found in virulent and avirulent sporulating cultures, respectively. A greater number of proteins found for the vegetative cells ordained a higher diversity of functional groups according to the Clusters of Orthologous Groups (COG) ontology (Figure 1d). Most of this abundance is dispersed among the household proteins. These included five cell metabolism enzymes, three protein folding and turnover mediators, and two translation elongation factors (for full protein names, see Table S1). Of the rest three proteins, two entries were assigned to the ‘Function unknown’ COG term and constituted a hypothetical protein and camelysin (CalY) M73 metalloprotease, and one was identified as NprB neutral protease (assigned to the ‘Amino acid transport and metabolism’ category by eggNOG based on sequence homology). Only two of these proteins, CalY and NprB, represent conventional *Bt* virulence factors.

Surprisingly, no three-domain Cry proteins were found in any sporulating cultures, except for the two spots from the avirulent strain, which were discarded due to discrepant motility features (data not shown). However, two ETX/MTX2-like proteins were found in both 800/3, and 800/3-15 spores, of which one was annotated as Cry15Aa, and the other one demonstrated high identity to the Cry60-like proteins from the NCBI Protein database. Five spots associated exclusively with the virulent spores were also found to contain a Cyt1-like protein. Moreover, the contents of two spots were annotated as an M6 metalloprotease highly similar to InhA1, and the contents of five spots were annotated as camelysin CalY, the former having been attributed to the ‘Defense mechanisms’ COG term. Additionally, an NprB protease was found exclusively in the virulent spores. Based on these data, we assumed that, notwithstanding a higher functional diversity of vegetative culture proteome, sporulating cells are more representative regarding the virulence determinants; therefore, we used proteomes of sporulating cells to compare different *Bt* serovars.

### 2.2. Spores of Serovars Israelenses, Darmstatdiensis, and Thuringiensis Demonstrate Distinct Patterns of Protein Presence

**Figure 2:**
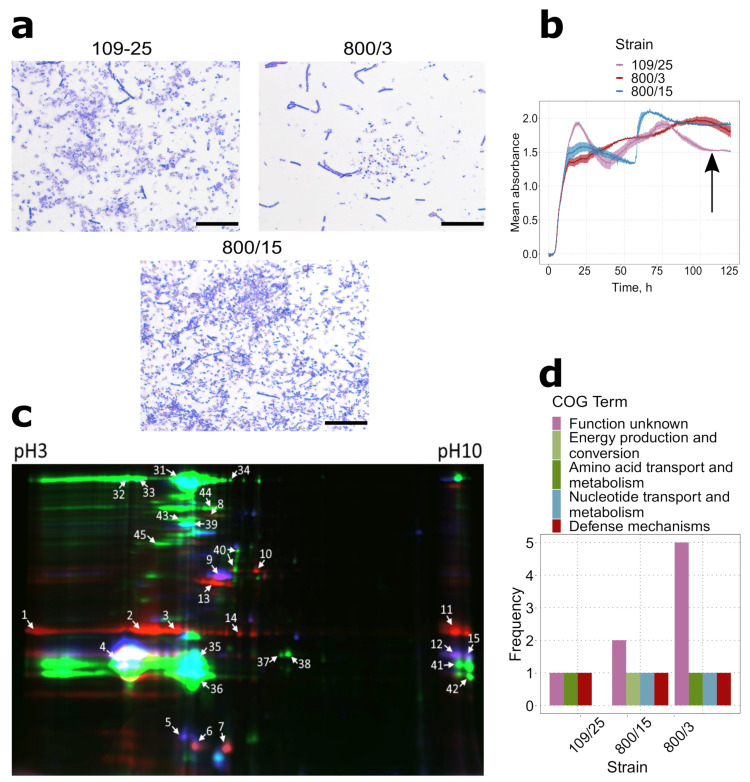
Proteomic signatures of *Bt* strains 109/25 (serovar *darmstatdiensis*), 800/3 (serovar *israelensis*), and 800/15 (serovar *thuringiensis*) (**a**) Microscope images of strain 109/25, strain 800/3, and strain 800/15 sporulating cultures All photos were taken at ×1000 magnitude in transmitted light. Scale bars are given as black rectangles and denote 20 μm. Parasporal inclusions were stained with Coomassie Blue. (**b**) Growth curves of strains’ 109/25, 800/15, and 800/3 cultures grown on T3 medium. The growth curve for strain 800/3 sporulating culture is the same as in Figure 1d. The black arrow marks the time of the spore culture’s protein extraction (**c**) 2D-DIGE image corresponding to the overlapping Cy2, Cy3, and Cy5 fluorochrome channels of *Bt* serovars spore proteomes. Red light channel indicates- proteins from strain 800/3, blue—strain 109/25 proteins, and green—800/15 proteins. (**d**) The COG term distribution among the proteins detected with ESI-MS. COG annotation was assigned to the reference sequences by sequence homology using eggNOG mapper.

We next selected two other crystalliferous strains, 109/25 and 800/15, to portray their spore proteome profile and compare it to that of strain 800/3’s. The choice of the strains was motivated by their belonging to the serovars broadly used for biologicals production, with strain 109/25 attributed to serovar *darmstatdiensis* and strain 800/15 representing serovar *thuringiensis*, respectively. Upon sporulation, both strains produce toxic crystals of conservative shape (Figure 2a,b), which demonstrate toxicity either to Coleoptera (strain 109/25) [28] or to Lepidoptera species (strain 800/15) [29]. As in the previous case, the sporulating status of the bacterial cultures was proved by both phase-contrast microscopy (Figure 2a) and growth curve reconstruction (Figure 2b).

By using the same proteomic protocol as applied to the *israelensis* cultures solely, we detected a total of 10 non-redundant proteins distributed between 30 fluorescent spots (Figure 2c, Table S2). Because most of the DIGE spots belonging to the strain 800/3 spores reproduced between the replicates, the identification results for this strain were transferred from the previous assay (see Figure 1 and Table S1). Of the proteins detected, five were found in strain 800/15, and only three proteins were detected in spores of strain 109/25. Such modest numbers correspond to the results previously obtained for serovar *israelensis* spore proteomes and are consistent with the general notion that spores are metabolically inactive and thus have a lower proteome abundance. Despite all the strains form crystal inclusions (Figure 1a and Figure 2a), three-domain Cry toxins were not detected in either of the strains again, and no ETX/MTX-like or Cyt-like toxins were found in spores of serovars *darmstatdiensis* and *thuringiensis*. The few identified proteins, however, represented bona fide virulence factors, such as CalY and InhA1 found in all three strains and NprB found in strains 800/3 and 109/25. The only notable exceptions were two proteins from strain 800/15 spores annotated as ATP synthase subunit beta and bifunctional metallophosphatase/5′-nucleotidase, respectively, which mapped to the cell metabolism-associated COG terms (Figure 2d).

Although very few proteins were identified in the spore proteomes, their connection to the strains’ virulent properties appeared more convincing than in vegetative cultures. Moreover, the presence of both shared and strain-specific genes allowed us to further compare the virulence factors repertoires and assign them to the serotyping positions of the strains.

### 2.3. The Distribution of Genes Corresponding to Products Identified with Proteomics Assay among Bt Genomes Does Not Support Strains’ Serovar Attribution

**Figure 3:**
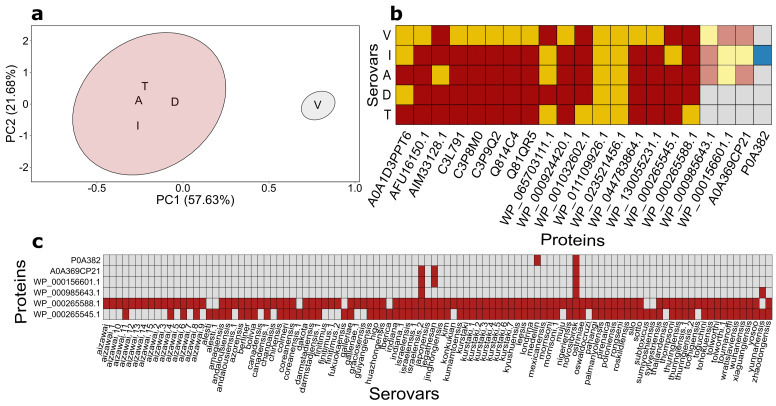
The distribution of genes related to proteins identified during the proteomic assay among analyzed samples and publicly available *Bt* genomes. (**a**) A k-means algorithm clustering results based on binarized proteomic data. ‘A’ and ‘I’ denote avirulent and virulent spores belonging to *israelensis* serovar, respectively; ‘T’ and ’D’ stand for *thuringiensis* and *darmstadiensis* serovars; ‘V’ signifies vegetative cells of strain 800/3. (**b**) The presence/absence of proteins and the corresponding genes among proteomes and genome assemblies with regard to their serovar’s attribution. Red color denotes a gene’s presence in a genome. Blue color indicates the presence of a protein in a proteome; yellow color is used if the protein/gene was found both in the proteome and genome belonging to identical serovars. Red and yellow colors’ intensity is proportional to the number of genomes in which it was detected. (**c**) The presence/absence of genes encoding proteomically-derived proteins among 104 *Bt* assemblies (15 core genes are not shown).

We then went on to visualize the difference between strains and stages of the bacteria life cycle using the principal component analysis (PCA) followed by k-means clusterization. Before conducting the analysis, the data were binarized according to the presence/absence of proteins within the proteomes (Table S3). The elbow method revealed that just two clusters were optimal to be used (Figure S1). Most of the samples (including virulent/avirulent derivates of the *israelensis* serovar as well as *thuringiensis* and *darmstadiensis* serovars) at the spore-forming stage grouped together, whilst vegetative bacterial cells formed a solitary cluster (Figure 3a). Using dots distribution at 2D-DIGE gel instead of only identified proteins increased PCA plot resolution and resulted in three clusters (Figure S2a,b) with sporulating cultures of virulent and avirulent *israelensis* strains clustered together separately from *thuringiensis* and *darmstadiensis* cluster. Unfortunately, the distribution of the dots at 2D-DIGE gels is difficult to use as a reliable method for strains’ serotyping (Figure 3b).

As it can be inferred from the resulting data, the discriminating status of proteins specific to particular serovars (or, at least, strains) remained uncertain due to the limited resolution of the proteomic approach chosen. A plausible way to test this association is to evaluate the presence of the respective genes in the available genome sequences. No genome assemblies have been produced for the used strains so far; however, representative genomes for each serovar as well as other major serovars are available at the NCBI Assembly database and thus can be used to test this hypothesis on a larger scale. To this end, the distribution of genes encoding proteome-inferred products among the quality-filtered 104 genome assemblies was dissected (Table S4). We first narrowed down the analyzed set of genomes to 3 serovars assayed by proteomics techniques and averaged the genes’ occurrence for assemblies belonging to the same serovars. As a result, 17 of 21 genes were pinpointed in all genome assemblies, 3 genes (WP_000985643.1, WP_000156601.1, A0A369CP21) were present in one *israelensis*-assigned genome out of 3, and the remaining gene (P0A382) was absent in any assembly.

Thenceforth, gene distribution was analyzed for all 104 assemblies. Of the genes analyzed, 15 were considered core domestic genes as their orthologs were found in every assembly (Figure S3, Tables S6 and S7). The remaining genes showed sporadic occurrence within the genomes. Notably, the dissimilarities corresponding to what was mentioned above were noticed for WP_000265588.1 and WP_000265545 (Figure 3c) co-occurrence for several serovars such as *aizawai*, *andalousiensis*, *canadensis*, *coreanensis,* and *galleriae*. The results obtained indicate that the usage of proteomically-inferred products in terms of their presence/absence in genomes does not corroborate the strains’ serovar attribution and cannot be used as a method for unambiguous serotyping independently of the mass-spec resolution. This observation stressed the need to reconstruct the *Bt* strains’ phylogeny based on either the well-known single-reference markers or phylogenomic inferences.

### 2.4. Pangenome-Wise Phylogeny Does Not Also Correlate with Serotyping Classification

#### 2.4.1. Pangenome Reconstruction

We performed a pangenome construction as a starting point for a phylogenomic study. We used a two-step Roary-based approach with the first run of Roary with 150 genomes downloaded from the NCBI assembly database with serovar attributions specified. After filtering the assemblies that did not pass the 50% threshold of common genes (for more details, consult Section 4.5.3), 104 genomes were kept for further examination. The resulting pangenome contained 57854 genes in total; 1965 of them were defined as core genes and 377 as soft core (Figure S4a–c).

#### 2.4.2. Pangenome-Derived Phylogeny

The phylogeny based on the pangenome could be constructed in two principal ways. First, we used the data on the presence/absence of accessory genes and cluster genomes with similar patterns. Howbeit, such an approach lacks the strength to reveal phylogenetic relationships and could only be considered a quick insight into the data. Thus, we additionally applied an ML-based (maximum likelihood) algorithm based on multiple sequence alignment of core genes.

The tree built on the presence/absence data was generated by Roary internal script with the FastTree utility. Subsequently, single nucleotide polymorphisms (SNPs) from the concatenated core genes’ alignment were retrieved to reduce the running time and memory usage during phylogenetic inference. The final pre-filtered alignment was 279 kb long, with a mean identity of 73.2%. The trees were characterized by high mean support values (91 and 86, respectively). It is noteworthy that the trees exhibited a noticeable topological similarity (90%). Importantly, we observed a marked discrepancy between serovar attribution and a pattern of clade formation within both trees. Genomes belonging to serovars *aizawai*, *galleriae,* and *tolworthi* split sufficiently in the presence/absence tree, whilst the core SNPs-based tree was characterized by a remarkable divergence for representatives of serovars *darmstadiensis* and *indiana* (Figure S5a,b). Therefore, we proceeded with tree construction emanated from flagellin sequences, which is considered the main antigen used for serotyping [4], to test whether it would dovetail with serotyping.

### 2.5. Flagellin-Based Phylogeny Is Remarkably Distinct from the Pangenome-Based Inference

Phylogenetic trees can be constructed based on amino acid or nucleotide sequences. The latter diverges faster than the former; therefore, we have used nucleotide sequences for tree building to separate flagellin sequences from close Bt strains. An additional rationale for the nucleotide sequence use was obtained by comparing the *gyrB* nucleotide- and amino acid-derived trees to the original Roary clusters (Figure S5c–e, Table S8). We then utilized 28 orthologous flagellin clusters produced by Roary as a basis (Tables S9 and S10). To verify that we have found all flagellin genes, we conducted an independent HMM-based (Hidden Markov Chain) search with *hag*-based hmm-models. The consistency between HMMER- and Roary-detected flagellin hits was examined. These two approaches displayed a striking resemblance (Table S11). Then, the flagellin ML-tree was build using the sequences related to the largest Roary cluster.

**Figure 4:**
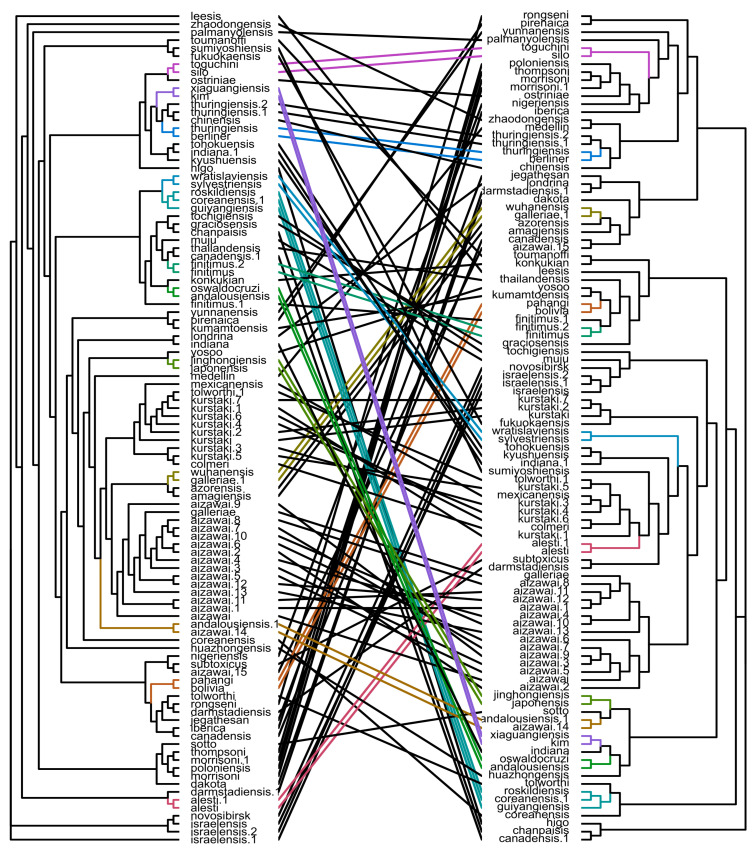
A tanglegram visualizing the differences in the topology of the core SNPs-derived tree and flagellin-based tree. Colored lines connect the subtrees with identical topology in both trees. Trees with supporting values and the lengths of nodes specified are available as Figure S5b,f.

Thereafter, we focused on comparing the topologies of pangenome- and flagellin-originated phylogenies. Remarkably, the phylogenies demonstrated a huge dissimilitude revealed both by a tanglegram tree representation (Figure 4) and quartet distance calculation (46%). Although some serovars were more closely grouped in the flagellin-based tree compared to the core SNPs-derived tree (e.g., *andalousiensis*, *coreanensis*, *indiana*), others, conversely, broke into separate clusters, such as *kurstaki* and *galleriae*. Thereupon, none of the approaches used is consistent with serovars’ attribution. Given that, we decided to explore a broader landscape of ANI(Average Nucleotide Identity)-based clusterization indifferent to trees’ topology. After applying PCA on the ANI matrix, two distinct serovar groups were obtained (Figure S6a,b, Table S12). While such an approach mainly tended to preserve all the representatives of a particular serovar in one cluster, notable exclusions were observed. For instance, assemblies with some serotype attributions, namely, *andalousiensis*, *canadensis*, *coreanensis,* fell into different clusters (Table S12).

### 2.6. Single-Loci and Genome-Wise Phylogenetic Trees Are Consistent with Each Other and Serotyping Classification at Different Degree

**Figure 5:**
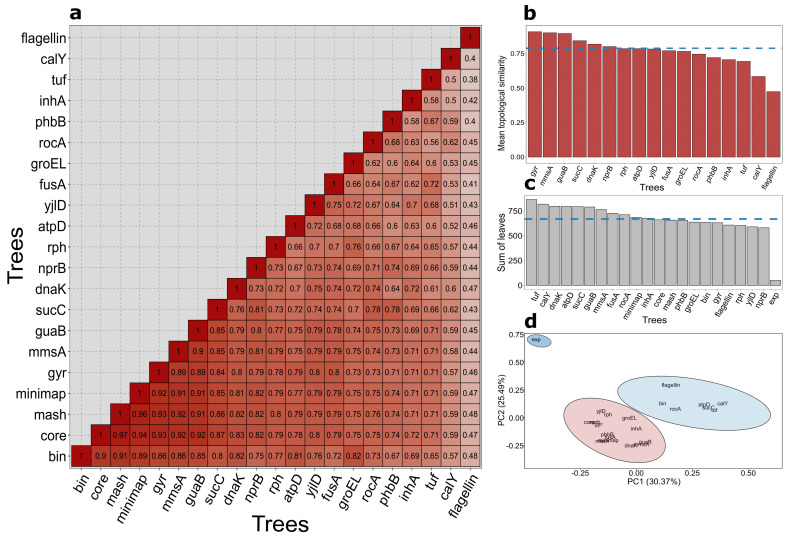
Topological comparison between trees and their relevance to serological classification. Most of the trees’ names refer to the respective gene identifiers; *bin* denotes presence/absence tree; *core* - core SNPs tree; *mash* and *minimap* - hierarchical clustering-obtained dendrograms based on mash and minimap2 output, respectively; *flagellin* - flagellin paralogs-emanated tree; *gyr* -the tree for the concatenated genes *gyrA* and *gyrB*. (**a**) Shown is a matrix depicting topological similarity (1-quartet distance) between phylogenomic and single loci-based trees. The intensity of the color is proportional to the identity. (**b**) Mean topological similarity for single-loci trees with reference phylogenomic trees. The blue dashed line represents the median value, and the same is in the next plot. (**c**) Plotted are the sums of the subtrees’ length pertaining to specific serovars. The blue dashed line represents the median value, and the same is in the next plot. Exp stands for the expected value (provided serovars’ representatives form monophyletic clades). (**d**) A k-means algorithm clustering results based on the number of leaves in subtrees comprising all representatives of the serovar. The solitary blue cluster comprises only the expected value.

Considering the results with gene absence/presence, core SNPs, and flagellin trees, we suggested verifying whether any of the single loci- or full genome-derived trees would finely reflect the immunological serovars’ classification. First, we scrutinized genomic data in a holistic manner using two categories of methods. The first one employed ANI, which was determined by calculating Mash distance. The second method implied that the whole-genome alignment was made with the minimap tool, followed by counting double the number of matches normalized by the sum of genome lengths. The results of both approaches were represented as matrixes, which were used for the hierarchical clustering procedure (Table S13, Figure S7a,b). Unquestionably, the obtained dendrograms are not distinctive phylogenies; notwithstanding, the samples’ grouping patterns could be informative and helpful in assessing ML-trees’ quality. The dendrograms were characterized by a strong topological resemblance between themselves and phylogenomic trees as well (Table S14, Figure 5a).

To assess whether any phylogeny of the core genes-encoding proteins identified with the proteomic approach describes the serotyping categorization more accurately than the flagellin’s one, we built phylogenetic trees for each of their loci. We also built a tree for concatenated genes *gyrA* and *gyrB*, which are known to be good phylogenetic markers for the *Bacillus* genus [30]. For each of the 15 genes found in all studied assemblies, nucleotide sequences were retrieved according to Roary-generated clusters (Table S10). After aligning and evaluating optimal evolutionary models, ML-trees were constructed (Table S8, Figure S5g–u). Trees obtained demonstrated immense diversity in quality, ranging in mean supporting values and the number of unique CD-HIT clusters, while all of them exhibited similar mean inter-sequence identity (94–99%, Table S8).

We then conducted a topology-comparing survey to identify single loci phylogenetic markers finely concordant with the phylogenomic approach. A representation of data as a heatmap allowed us to reveal distinctive patterns (Figure 5a). While most trees were remarkably concordant (75% mean similarity, Table S14), the flagellin-derived tree vastly differed from other trees (44% on average). We also calculated the average similarity score between every loci-evolved tree and 4 references (trees based on binary presence/absence of accessory genes, core SNPs, mash distance, and full genomes’ pairwise identity, respectively, Figure 5b), which clarified details. Of all the cases, widely adopted usage of the gyrase sequences for *Bt* strains delineation was also the most appropriate variant reflecting phylogenetic relationships (with a score of 91%). Notably, we revealed other markers with pretty similar representative properties, namely, *mmsA* (90.03%), *guaB* (90%), and *sucC* (85%).

To draw parallels between serotyping classification and the phylogenies, for each serotype, we counted the number of leaves in the minimal subtree containing all serotype representatives and simply summed up the lengths of these subtrees (Figure 5c). All phylogenies exceeded the expected sum by more than ten times (Table S15). Peculiarly, not the flagellin, but *nprB* displayed the lowest score (582), which still was quite similar to flagellin ones (609). As simple summation ignores the dissimilarities for specific serovars, we applied PCA to consider each serotype separately (Figure 5d), and it was characterized by three clusters optimally (Figure S8). The results generally corroborated the summation method except for the core SNPs-derived tree and the *yjlD*-based tree being closer to the expected value. Interestingly, the phylogenies used fell into two separate clusters: one included core SNP, *inhA*, *gyr*, *dnaK*, *groEL*, another comprised of presence/absence, flagellin, *calY,* and other core genes. Nevertheless, all of the points corresponding to different phylogenies did not fall into one cluster with the expected case.

Finally, we decided to test whether 3-D cry toxins’ distribution reflects the discernment between serovars. As a result, we found that different toxin sets are non-homogenously distributed among representative of a specific serovar (e.g., for serovar *kurstaki*; *aizawai*, *alesti*, etc., Table S16), and we also observed that lack of Cry toxins’ genes is quite common for multiple strains (*aizawai*, *darmstadiensis*, *morrisoni*, etc., Table S16).

## 3. Discussion

In total, the proteomic protocol resulted in the identification of 21 non-redundant proteins dispersed across 45 distinct DIGE spots. The distribution of the identified proteins between the spore and vegetative culture proteomes is consistent with the principal notions on the metabolic differences between the respective stages of *Bacillus* lifecycle. Most of the proteins detected in the vegetative cells’ culture are involved in constitutive cell metabolism. Unlike this, two metabolic enzymes were detected in spore proteomes, but their roles in the sporulating cultures have not been revealed so far.

A major drawback of the technique used lies in the absence of any identified three-domain Cry proteins. All three strains have been previously demonstrated to possess Cry-encoding genes [26,27]. A plausible explanation for this lack comes from low solubilization of Cry toxins in traditional protein extraction buffers [11], which may require additional rounds of protein solubilization [10]. However, Cyt1-like toxins have been detected in strain 800/3′ proteome, and ETX/MTX2-like Cry toxins were found in both virulent and avirulent spores of serovar *israelensis*. The presence of non-three-domain Cry toxins in avirulent strain’s 800/3-15 proteome is somewhat peculiar given that it does not produce crystalline inclusions. It is possible that the avirulent strain is, in fact, capable of crystal toxin production but is impaired in crystal assembly. The production of parasporal inclusions is known to be a complex process involving the activity of different auxiliary proteins, such as proteases [31,32] and molecular chaperones [10,33,34], thus leaving room for speculations on the real reasons underlying the loss of toxicity in 800/3-15. Taken together, the implemented approach appears ineffective for large scale proteome assays. It should be noted that some other gel-driven proteomic assays with a similar experimental layout revealed a larger number of non-redundant proteins [11,35,36,37], including Cry toxins [38,39]. However, other studies exploiting multi-probe fluorescent DIGE have stated the numbers of identified proteins similar to what was obtained in the current study [40,41,42,43]. The applied methodology itself could explain relatively small resultant figures; mainly low sensitivity and the incompleteness of the underlying databases contribute to limitations of gel-based proteomics.

For 21 non-redundant proteins, only 6 corresponding coding sequences were found to vary in terms of presence/absence among all the genomes surveyed. Not exclusively the obvious hits pertaining to domestic genes, such as identifiers relating to chaperones, elongation factors, and enzymes of primary metabolism, were considered core loci. However, several orthologs of virulence determinants (*calY*, *inhA1*, *nprB*) were observed in all the genomes as well. The ubiquity of genes encoding for CalY and InhA1 was somewhat unexpected, given that the respective names explicitly appear in a small fraction of genome annotations [9]. This circumstance underpins the urge for proper annotation of the deposited genomes, as well as for the thorough pangenome-wise assessment of virulence determinants’ distribution among the *Bt* strains. InhA1 was demonstrated to play diverse functions in the *Bt* pathogenesis, including host’s humoral immune response alleviation [44] and the enhancement of the pore formation in the intestine cells [45], and CalY is involved in extracellular matrix digestion [46] and, apparently, Cry toxin activation [31,32]. Among the proteins identified, another closely associated with virulence was neutral protease B (NprB), which also appeared to be encoded in all the genomes analyzed. This peptidase carries out processing PapR, which acts in the PlcR-PapR quorum sensing system regulating the expression of virulence-associated genes in *Bc* complex species [47,48]. Alternatively, an Npr599 protein *B. anthracis* was shown to cleave the murine exoproteome components, thus serving as a *bona fide* virulence factor [49]. Moreover, the NprB production itself is promoted by the PlcR transcription regulator at the pre-spore-forming stage [50]. Together with InhA1, NprB (sometimes designated as NprA or Npr599) constitutes 60 to 80% of the virulent *Bacillus* secretome, which was considered a differentiative marker for assessing pathogenic activity [35,49,51]. It has also been demonstrated to undergo positive selection in pathogenic *Bacillus* cereus group species [52]. The ubiquity of the respective genes is noteworthy considering the problem of *Bt* pathogenicity mechanisms but contributes little to serovar delineation. In fact, of all the virulence factors spotted, only the ETX/MTX-2 like Cry toxins differ in their presence among strains considering their structural diversity. Taking into account that the proportion of unique proteins constitute at best half of the total spots selected (21/45) and that most of these spots comprised duplicate proteins, we propose that either the proteome-driven approach lacks in sensitivity to capture the full repertoire of virulence factors, or that the selected strains did not differ in this regard despite their attribution to different serovars.

Indeed, the serotyping technique, notwithstanding its wide use, suffers from several drawbacks. First, serotyping is obviously inapplicable for the characterization of non-motile and autoagglutinating isolates [53]; it also may lead to spurious false positives when assessing acrystalliferous strains genetically close to other *Bacillus* species [54]. Then, testing the agglutination with all the antisera is both expensive and cumbersome, thus being a privilege of few laboratories possessing all the antisera varieties [8]. To obviate these difficulties, several genetically oriented approaches have been put into practice, such as M13 fingerprinting [55], repetitive extragenic palindromic polymerase chain reaction (Rep-PCR) fingerprinting [53], multilocus enzyme electrophoresis (MLEE), and random amplified polymorphic DNA (RAPD) profiling [56]. The robustness of these methods is undermined by the fact that the serovar attribution does not actually reflect any genetic similarity or evolutionary relations between strains [8]. In the past two decades, phylogeny reconstruction assays based on the single reference loci sequences have been undertaken to justify the existing systematics. Because the 16S rDNA sequence often fails to discriminate strains of *Bacillus cereus* species group other than *B. anthracis* [57,58,59,60], several protein-encoding loci have been proposed to serve as phylogenetic markers. Since flagellin is a primary antigen used for *Bt* serotyping, the most genuine approach suggests using *hag* locus encoding for flagellin as a reference one. Despite this, flagellin-derived phylogenies did not corroborate the monophyletic status of the distinguished serotypes [8,61]. Moreover, *hag* loci are prone to duplicate within the genomes, and the resulting paralogs impede proper phylogeny inference [61]. Another example is *gyrB* gene encoding for gyrase beta subunit, which was shown to delineate *Bc* species at a level of accuracy compared to that of DNA-DNA hybridization [30,62], and the *aroE* gene encoding for shikimate dehydrogenase [62] demonstrates similar yet slightly lower discriminative properties. In spite of the controversies around the applicability of flagellin encoding-sequences, serotype determination based on the respective phylogeny reconstruction is still used in the *Bt* studies [63,64,65]. In the aforementioned studies, the location of surveyed microorganisms on the phylogenetic trees is considered an argument for asserting the group. Moreover, occasionally even the 16S rDNA sequence is treated as proof for attributing serovar identity [66]. In the present work, we attempted to go further than single loci-restricted phylogeny and undertook pangenome-wise analysis accordingly.

The pangenomes of different *Bacillus* species as well as the *Bc* species complex have frequently been subjected to reconstruction attempts and have been evaluated to be mostly open, e.g., having its accessory component larger than the core one [67,68,69]. Speaking of *Bt* solely, its pangenome has previously been shown to be open as well [67,70], with a consistent increase in size with each strain added [70]. The pangenome-derived data, such as the core alignments and the distribution of accessory genome components, as well as the usage of genome-wise comparative analysis, could come up with comprehensive phylogenomic relationships capable of delimiting individual genomovars considering sporadic alterations in genomic architecture. The so-called Feature Frequency Profiles (FFP) method implicating SNPs’ acquisition across the *Bacullis* genome thoroughly outstripped single loci-emanated phylogenies in terms of precision and efficiency, preserving the monophyletic status of *B. thuringiensis*, *B. anthracis*, and *B. cereus* [71]. Yet another recent study utilized SNP-based phylogeny to dissect close evolutionary interconnection in *Bti* strains [15]. Aside from *Bt*, the application of SNPs for phylogeny reconstruction exhibited fine genomic clarification in other bacterial species, including *Escherichia* [72], *Burkholderia* [73], and *Bacillus cytotoxicus* [74]. Another frequently applied technique suggests calculating whole-genome metrics, Average Nucleotide Identity (ANI) [75]. ANI-based approach assisted in the determination of new *Bacillus* isolates from the gallinaceous feces [76], enabled to disclose different clades within *Bacillus cytotoxicus*. An approach similar to ANI, namely genome BLAST distance phylogeny, has also been applied to derive the full-genome phylogeny of the *Bc* species complex [60]. Importantly, tracing average nucleotide identity uncovered that *B. thuringiensis*, in fact, diverges and comprises two separate genomovars, namely, *B. thuringiensis* gv. *thuringiensis* and *B. thuringiensis* gv. *cytolyticus* [13]. In the current study, the mean ANI value (95.8%) lies above the conventional threshold for genomospecies, which is originally defined as 95% [75]. The minimum value observed (92.6%) slightly exceeded the empirically evaluated threshold for *Bacillus* genomovars (92.5%, [77]). However, based on PCA results, two separates clusters were obtained with mean ANI values of 97.2 and 95.5, respectively, indicating that the analyzed assemblies referred to diverse genomospecies. Since for some serovars, their representatives split between these clusters, it could indicate either erroneous serotyping or the inconsistency between genomic evolutionary relationships and serovars’ attribution.

The dendrograms based on clustering genome-similarity matrices (both mash- and minimap2-derived) as well as phylogenetic trees (binary presence/absence, and core SNPs) displayed substantial topological similitude. It indicates that all the methods could provide elaborated genome-wise phylogeny, and single loci-derived trees more or less accurately reflected them except for the flagellin-based tree, which topology demonstrated the immense difference. Noteworthy, none of the phylogenies reconstructed agreed with serotypes. Thus, flagellin-derived phylogeny not only failed to discriminate serotypes but also did not reflect the genomic structure of *Bt* strains. Our results are in agreement with previous findings that the phylogenetic position of bacterial genomes did not illustrate an evident correlation between phenotypic traits [78].

Though single loci phylogenies built failed to discriminate serotypes, but at PCA plot, they fell into two categories, closer to either the core SNP tree or presence/absence tree. This observation might indicate that even within virulence factors, there are two groups of genes, which evolve more like core genes or as an accessory part of the genome.

The distribution of 3D cry toxins also failed to discern between serovars as both the absence of toxins and diverse combinations within one serotype were observed. These two instances could be explained by the location of genes encoding these toxins. As most of *Bt* toxins’ genes are characterized by plasmid location [79], it is no wonder that relatively often, *Bt* strains could lose these plasmids and become acrystalliferrous afterward [80]. Furthermore, extrachromosomal elements tend to participate in recombination events, both homologous and non-homologous, which can form new plasmids with different combinations of toxins even within one strain [81,82].

Taken together, the use of more than one locus, as in the case of multilocus sequence typing (MLST) [20,78] or phylogenomic approach [71,83], shows that the distribution of phenotypic features among the strains of *Bc* species, including the *Bt* serological groups, does not confine to any of the established phenotypic classifications. In the present work, none of the phylogenetic inferences virtually supported the serotyping-based division, which further urges the re-evaluation of the established classification and adherence to the large-scale phylogenetic approaches. A possible alternative could be based on phylogenomics’ principles implementing the tracking genome dynamics in an evolutionary context. In this instance, an appropriate method is grouping Bt genomovars based on the location of syntenic blocks among the genome.

## 4. Materials and Methods

### 4.1. Bacterial Strains and Growing Conditions

Virulent strains of *B. thuringiensis* 800/3 (serovar *israelensis*), 109/25 (serovar *darmstadiensis*), and 800/15 (serovar *thuringiensis*), as well as avirulent strain 800/3-15 (serovar *israelensis*), were used in the study. To obtain a vegetative culture, the strains were grown on Luria-Bertani (LB) agar Petri plates for 15–18 h. To obtain the culture that had completed sporulation (till microscopic analysis detected only spores and protein crystals), the strains were incubated on T3 [84] agar Petri plates for 5 days at 30 °C.

CLARIOstar Plus (BMG LABTECH, Germany) was used to plot the growth curves. 96-well plates were filled with 200 μL of T3 and LB liquid medium inoculated with bacteria strains (four replicates for each strain on each medium). Equivalent volumes of sterile media were used as blank samples. Measurement of optical density was carried out for 5 days with periodic shaking of the plate and maintaining the temperature of + =30 °C. Resulting data were averaged over four replicates per each strain and visualized using ggplot2 package [85] v3.3.2 for R programming language (ref.) v3.6.3 with the error bars denoting standard error of the mean.

Microscopy assays were performed using a phase-contrast microscope (1000× magnification) on 1–5 days of their growth on T3 agar plates to register the beginning of sporulation and the presence of crystal inclusions. For evident crystal determination, Coomassie Blue staining was used [86]. Slides with fixed bacterium were immersed in the dye (0.133% Coomassie Blue stain in 50% acetic acid) for 2 min and rinsed with distilled water.

### 4.2. Protein Extraction, Two-Dimensional Fluorescent Difference Gel Electrophoresis, and Protein Mass-Spectrometry

For protein extraction, bacteria were centrifuged, washed, and resuspended in a lysis buffer (7M Urea, 2M Thiourea, 4% CHAPS, 25 mM Tris pH 8.2) in approximate 10 volumes of lysis buffer to 1 volume of cells. Cell homogenization was conducted by sonication with QSonica Q125 sonicator (Newtown, Connecticut, USA) at 30% amplitude for ten seconds. The sonication step was repeated five times, with test tube contents having been shaken gently and the sonicator rod sterilized between the rounds. Protein concentrations were measured by the absorbance at 280 nm NanoDrop ND-1000 spectrophotometer (NanoDrop Technologies), Bradford assay, and PAAG-electrophoresis [87].

Prior to two-dimensional electrophoresis, samples were conjugated with Cy2, Cy3, or Cy5 dyes (Lumiprobe, Hunt Valley, MD, USA) in a proportion of 400 pM of a dye to 50 μg of total protein. Samples were conjugated on ice for 40 min; then, the reaction was stopped by the addition of 10 μM L-lysin for 15 min. The samples, conjugated with different dyes, were mixed together and with dithiothreitol (up to 100 mM) before electrophoresis. Due to the presence of highly abundant proteins, two replicates with different protein amounts were analyzed—approximate 75 and 150 μg of total protein were loaded into each IPG-strip (7 cm, pH 3–10; BioRad, Berkley, CA, USA) by overnight passive rehydration at room temperature. Each gel contained samples of total lysates of three strains of bacteria. No less than two technical replicates were done for each biological replicate and for each protein concentration with Cy-dyes swap. Isoelectric focusing (IEF) was performed using the Protean IEF Cell (BioRad) according to manufacturer recommendation (10 000 V/h, end voltage 4000 V, maximal current 50 mA per IPG-strip, rapid voltage ramp, 20 °C). Then IPG-strips were consequently incubated in two equilibration buffers (6 M urea, 2% SDS, 20% glycerin, 0.375 M tris, pH 8.8) for 15 min with either 2% dithiothreitol and 2.5% iodoacetamide. After equilibration discontinuous electrophoresis in 14% PAAG was performed (BioRad; Laemmli, 1970). Different Cy-dyes were visualized using the Typhoon FLA 9500 laser scanner (GE Healthcare, Chicago, IL, USA). After the Cy-dyes visualization, the gels were stained with Coomassie G-250. The protein spots of interest were excised from stained gels in no less than two technical replicates and identified following the “bottom-up” approach described earlier [24,25].

The selected gel fragments were cut to pieces with 1 mm^2^ approximate size, destained with 50% acetonitrile in 25 mM Tris (pH 8.2), dehydrated with 100% acetonitrile, and rehydrated with proteomics grade bovine trypsin solution (20 ng/μL, 25 mM Tris, pH 8.2, Sigma) on ice for 60 min. Excessive trypsin solution was removed, and the gel was covered with 30 μL of 25 mM Tris (pH 8.2). Tryptic digestion was performed at 37 °C overnight. Tryptic peptides were eluted with 50% acetonitrile/0.1% formic acid and analyzed using HPLC coupled with tandem mass spectrometry (Agilent 1260 coupled with ESI-Q-ToF Agilent 6538, Agilent Technologies, Santa Clara, California, CA, USA). The gradient elution method was 0% B phase to 60% B phase for 45 min and further to 100% B phase for 10 min. B phase was 90% acetonitrile with 0.1% formic acid, A phase was 5% acetonitrile with 0.1% formic acid; the flow rate was 20 μL/min; the column was Zorbax B-C18 5 μm grain, 80 Å pores, 150 × 0.5 mm (Agilent Technologies). The mass spectrometry was performed in positive ion mode with auto MS/MS collection in precursor mass range 100–3200 Da.

Protein identification by MS/MS-spectra was performed using Agilent Spectrum Mill MS Proteomics Workbench Rev B.04.00.127 in the mode ‘Identity’ against the Swiss-Prot database (taxonomy: “Bacteria [2]”, September 2020, 334639 sequences) and protein sequences from Identical Protein Groups Database (https://www.ncbi.nlm.nih.gov/ipg/, accessed on the 3th September 2020), related to the *Bacillus* species (312044 sequences). The precursor mass tolerance was set to ±20 ppm. The validation procedure of identified proteins was performed with a minimum protein score of 15 and a peptide false discovery rate (FDR) for validated proteins of 1%. The resulting protein lists were manually checked for duplicates and ambiguously annotated spots. Proteins annotated as closely related accessions were assigned a common annotation, and proteins demonstrating discrepancies between the observed and predicted values of molecular weight and isoelectric point were excluded from the further analysis.

### 4.3. Protein Functional Annotation

The reference protein sequences were downloaded from NCBI Protein [88] and Uniprot [89] databases using a custom Python script implementing Biopython v1.73 [90] functionality. The presence of 3-d Cry toxins was checked with CryProcessor [91]. Functional annotation of the obtained sequences, including the Cluster of Orthologous Genes (COG) attribution assessment, was carried out with eggNOG standalone tool v2.0.1b-2-g816e190 [92].

### 4.4. Flagellin Sequence Search in the Genomic Data

Flagellin gene sequences were annotated in the *Bt* genomic data using a hidden Markov model (HMM) approach implemented in HMMER v3.3.1 [93]. 30 *hag* gene sequences obtained by Xu and Côte [60] were first aligned using MAFFT [94] v7.453 in careful mode (using ‘--localpair’ option and having ‘--maxiterate’ parameter set to 1000), and the resulting alignment was compressed to HMM using hmmbuild utility. The obtained model was used for flagellin genes search using hmmsearch utility. For the sake of sanity check, HMM search results were manually revised by alignment to NCBI Nucleotide database using BLASTn utility [95]. To exclude sequences unrelated to the flagellin paralogue family, e.g., flg basal hook protein genes, an additional E-value cutoff equal to 1E-10 was introduced. The notions underlying the further selection of representative flagellin gene sequences for phylogeny reconstruction are described in Section 4.6.

### 4.5. Bt Genomes Acquisition and Pan-Genome Reconstruction

#### 4.5.1. Data Acquisition

The assemblies referring to *Bacillus thuringiensis* were obtained from the NCBI Assembly database (Table S4). Only those with serovars specified remained. We also filtered out the genomes not comprising full-sized flagellin genes (containing the corresponding protein product less than 100 amino acids long). Finally, we performed the quality-control selection based on the number of genes in the pangenome.

#### 4.5.2. Gene Presence Analysis

Proteomes of bacterial cells with serovars attributed (*israelensis*, *thuringiensis,* and *darmstadiensis*) at the sporulation stage, as well as different stages of *israelensis* serovar, were compared with PCA (Principal Component Analysis). For proteins identified, we assigned ones or zeroes dependent on their presence/absence in the sample accordingly (Table S3). The data being sparse, it was transformed into the dissimilarity matrix using Bray-Curtis distance [96] with vegdist function from the vegan v2.4-2 [97] package for R programming language. Next, the ‘pcoa’ (Principal Coordinate Analysis) function from ape v5.4-1 [98] R package was applied. The samples were then clustered with the k-means algorithm (nstart = 25, iter.max = 1000). The optimal number of clusters was evaluated with the elbow method [99] by depicting with-in-Sum-of-Squares (WSS). Clustering results were subsequently visualized via the ‘autoplot’ function from ggfortify v0.4.11 [100].

The presence of proteins in the assemblies was obtained through Diamond v2.0.4 [101] blastp in sensitive mode (‘--more-sensitive’) with ‘--max-target-seqs’ parameter set to 1. Because of the urge to determine the identity cutoff for Diamond, the reference sequences of inhA virulence factors were compared via calculating a Distance matrix using Biopython with the minimum and maximum similarity of 67% and 84%, respectively (Table S5); the minimum value (67%) was set as an identity threshold for Diamond. The best hits were aggregated according to the following scheme with the custom python script. Initially, the lowest e-value hits were selected. If several hits were found, the most similar to references were preferred. Eventually, if needed, the longest sequences were retained.

#### 4.5.3. Pangenome Reconstruction

Pangenome analysis was carried out via Roary v3.11.2 [102]. Before creating the pangenome, we attempted to re-annotate the assemblies with a uniform database, as recommended in the Roary manual. The FASTA-files from the IPG (Identical Protein Groups) database for *Bacillus thuringiensis*, *anthracis*, and *cereus* were concatenated and applied as the source of protein sequences for Prokka v1.14.5 [103]. Nonetheless, default annotations outperformed Prokka-generated ones in accuracy and completeness, e.g., containing fewer hypothetical proteins and erroneously identified CDS; hence, the initial annotations remained unchanged.

Roary was launched with a 95% identity cutoff for blastp in alignment mode, allowing to retrieve core genes’ alignments. We also increased the maximum number of clusters to 100,000 as *Bacillus* species are characterized by the acquisition of non-essential genes resulting in genomic variability [15]. Forasmuch as way too many genes in a pangenome may indicate the inappropriate quality of the underlying data and diminish the analysis’s predictive accuracy [104], we proposed a simple metric somewhat by analogy with N50. All genes in each assembly were ranged in ascending order according to their presence among the samples. We then calculated the number of genes contained in more than half of the genomes and pitched upon the assemblies for which that sum exceeded 50% of genes accordingly.

### 4.6. Phylogeny Reconstruction

Sequences (either protein or nucleotide) in FASTA-format were aligned with MAFFT v7.471 [94] in localpair mode with 1000 iterations for greater accuracy. Optimal evolutionary models were selected based on the BIC (Bayesian information criterion) values obtained via modeltest-ng v0.1.6 [105] in maximum likelihood topology mode. After that, maximum likelihood trees were reconstructed with raxml-ng v1.0.1 in all-in-one mode with 1000 bootstrap replicates. Mean support values for obtained trees were calculated with the Python script. Trees were visualized via the ggtree [106] v3.11 R package. The underlying multiple sequence alignments were quality-checked by assessing the mean identity via two approaches. After running the CD-HIT program v4.8.1 [107] with a 100% clustering threshold and the word size of 5 letters, the number of unique clusters was calculated. Besides, the mean blast-like sequence identity was evaluated via a custom Biopython-based script. In brief, combinations (regardless of the order) of two elements from the sequence set were aligned in global pairwise mode, and the percentage of matches was calculated afterward. Finally, the mean identity score was determined.

We proposed four types of trees as possible phylogenomic references: binary presence/absence, core SNPs, mash distance, and full genomes’ pairwise identity. The binary presence/absence tree of accessory genes was generated by Roary during pangenome construction. To receive core SNPs, we used SNP-sites v2.5.1 [108] on Roary-derived core genes’ alignment.

We also reconstructed phylogeny based on well-established markers such as gyrase subunit beta and flagellin sequences as well. *Bacillus thuringiensis* is known to possess several flagellin-coding genes (*hag*/*fla*), and no elaborated approach to pick the suitable gene exists. Hence, we fetched orthologs generated by Roary with a custom Python script. Concisely, if a genome contained more than one *hag*/*fla* gene, the gene pertaining to the largest cluster was preferred. If the sequence was too short (probably, being a gene fragment), the gene referred to the following cluster was selected. Finally, we reconstructed trees based on core genes (found in all the assemblies) encoding proteins identified with the proteomic assay. The corresponding Roary-generated clusters were used. Most of them formed a single orthologous group, and if genomes contained paralogs, the longest sequence was preferred (Table S10).

The mash distance-based matrix was constructed via a custom python script for parsing Mash v2.2 [109] output launched with a k-mer size of 21 and a sketch size equal to 100,000. To calculate the pairwise alignment-based metrics, we used minimap2 v2.17 [110] in assembly to assembly mode (-a asm5), disabling secondary alignments (‘—secondary = no’). Reference was determined in line with the assembly level. Initially, the most completed assembly in the pair was selected (e.g., the chromosomal level was preferred over contig). If levels were the same, we assigned as reference the assembly comprising fewer FASTA-records (contigs, scaffolds, etc.). The identity between genomes was calculated as:

(1) $$id=\frac{m*2}{g_{1}+g_{2}}$$

where *id* is identity, *m* denotes the total length of matches, while *g_1_* and *g_2_* stand for genome lengths, respectively.

Notwithstanding that secondary alignments were disabled, many overlapped mappings still were observed; thus, intervals merging was required to correct the possible identity percent exceeding 100%. As the minimap2-derived mappings lacked specific matching positions—only the total number was provided—we could not merely intersect intervals without losing precise information about matches. We hence decided to calculate the proportion of each interval in the union:

(2) $$id_{u}=\frac{id_{1}*\left(l_{1}-l_{i}\right)}{l_{u}}+\frac{id_{2}*\left(l_{2}-l_{i}\right)}{l_{u}}+\frac{\frac{l_{i}}{l_{u}}*\left(id_{1}+id_{2}\right)}{2}$$

where *id* denotes the BLAST-like identity of the interval (indices 1, 2 refer to the initial intervals, *i* means intersection and *u* defines union), and *l* stands for the length.

Both mash- and minimap-derived results were gathered and transformed into a matrix using a custom python script implementing NumPy v1.17.2 [111] and scikit-learn [112] v0.23.2 Python modules. Matrices obtained were then subtracted from 1 to produce distance matrices. To obtain trees, a hierarchical clustering procedure was carried out via the ‘hclust’ function from stats v3.6.2 incorporated as a default package for R by using the “complete” agglomeration method. The resulting dendrograms were converted into a tree of class “phylo” via ape v5.4-1 package and subsequently saved in Newick format. To depict the heatmaps, the corresponding matrices were arranged according to clusters’ order. The optimal number of clusters was selected using the silhouette function from the default R package cluster v2.1.0. The ANI-based matrix was also used to analyze the clusterization patterns between serovars based on their nucleotide identity. The procedure was carried out via the PCA method as described in Section 4.5.2.

All the reconstructed trees were topologically compared via tqDist v1.0.2 [113]. ‘quartet_dist’ utility. Calculated quartet distances were presented as a matrix that was visualized with the ggplot2 package. Flagellin- and core SNPs-derived trees dissimilarity was depicted as a tanglegram using the dendextend v.1.14.0 R package [114]. Before that, dendrograms were untangled with the ladderize method. So far as ML-trees are not ultrametric, thus unable to be converted to a dendrogram, the ReadDendrogram function from DECIPHER v.3.11 [115] was applied.

Finally, we assessed the serovars’ attribution. To this end, we proposed a simple approach that implies counting the number of leaves in a subtree containing all representatives of each non-singleton serovar attributed to the assemblies. For this purpose, we implemented the ‘get_common_ancestor’ function from the ete3 toolkit [116] v3.1.2 Python module. Next, the sum of the subtrees’ length pertaining to specific serovars was considered a simplistic integral score. Besides, the respective serovars-related data were analyzed with the PCA analysis and a concomitant k-means clusterization.

We applied CryProcessor in ‘*fd*’ mode on translated sequences of the assemblies’ genes to check the consistency between serovars’ attribution and the spectrum of insecticidal toxins. After CryProcessor [91] launch, we summarized the results obtained with a custom Python script (Table S15).

## Data Availability

All scripts used in this work are available at https://github.com/lab7arriam/IJMS_2020/. All data are available as supplementary materials.

## Supplementary Materials

The following are available online at https://www.mdpi.com/1422-0067/22/5/2244/s1, Table S1: Proteins identified in virulent spores and vegetative cells of strain 800/3 and avirulent spores of strain 800/3-15; Table S2: Proteins identified in virulent spores of strains 109/25 (serovar *darmstatdiensis*), 800/15 (serovar *thuringiensis*), and 800/3 (serovar *israelensis*); Table S3: Binarized data of protein presence obtained with proteomics approach. Zero stands for absence, while one denotes the presence of the protein. For each protein accession, the respective COG groups are given; Table S4: Properties of assemblies used in the study. The GenBank accession, assembly type, strain, and serovar attribution are presented; Table S5: A distance matrix representing the identity between reference *inhA* sequences; Table S6: Genes encoding proteomically detected proteins among the assemblies at a 67% threshold for Diamond; Table S7: The list of gene identifiers found in all analyzed assemblies (core genes); Table S8: Properties of the reconstructed phylogenetic trees (appropriate evolutionary model, mean identity and supporting values, number of unique CD-HIT clusters); Table S9: Roary-generated gene presence/absence results among the reconstructed pangenome; Table S10: Roary-produced clusters for the single-loci (flagellin and core proteins) phylogeny reconstruction; Table S11: Flagellin paralogs obtained from Roary launch and HMM-search; Table S12: The results of k-means clusterization of serovars using ANI-based matrix; Table S13: Similarity matrix between assemblies derived from calculating mash-distance and genome pairwise comparison with minimap2; Table S14: Topological quartet distance between trees; Table S15: Lengths of subtrees containing representatives of *Bt* serovars; Table S16. The list of 3D Cry toxins revealed with CryProcessor for each assembly, respectfully; Figure S1: The optimal number of clusters for the k-means clustering of the binarized DIGE data using the elbow method; Figure S2: The PCA and k-means clustering results obtained by utilizing all the protein spots found in 2D-DIGE gels; Figure S3: The distribution of gene encoding proteins identified among the *Bt* assemblies; Figure S4: Visualization of Roary-obtained pangenome reconstructed on 104 pre-filtered *Bt* assemblies; Figure S5: All phylogenomic and single-loci phylogenetic trees based on *Bt*-genomes; Figure S6: The results k-means clustering procedure based on ANI matrix; Figure S7: Heatmap visualization of clusters based on mash-distance and mean genome identity; Figure S8: The optimal number of clusters for the k-means clusterization of the data based on subtrees’ length containing all representatives of each serovar using the elbow method. All scripts used in this work are available at https://github.com/lab7arriam/IJMS_2020.
