# Supplementary material for: The Distribution of Several Genomic Virulence Determinants Does Not Corroborate the Established Serotyping Classification of Bacillus thuringiensis
Source: Int J Mol Sci. 2021 Feb 24;22(5):2244. doi: 10.3390/ijms22052244 (PMC7956386; doi:10.3390/ijms22052244)
Supplement: Supplementary file 1 [file ijms-22-02244-s001.zip › Supplementary_figures.pdf]

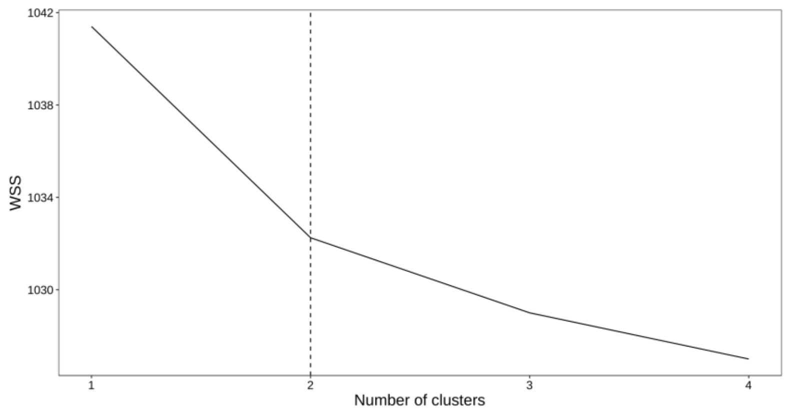

Figure S1. The optimal number of clusters for the k-means clusterization of the binarized 2-DIGE data using the elbow method. The dashed line represents the number selected (2) depicted in Figure 3a of the main text.

a

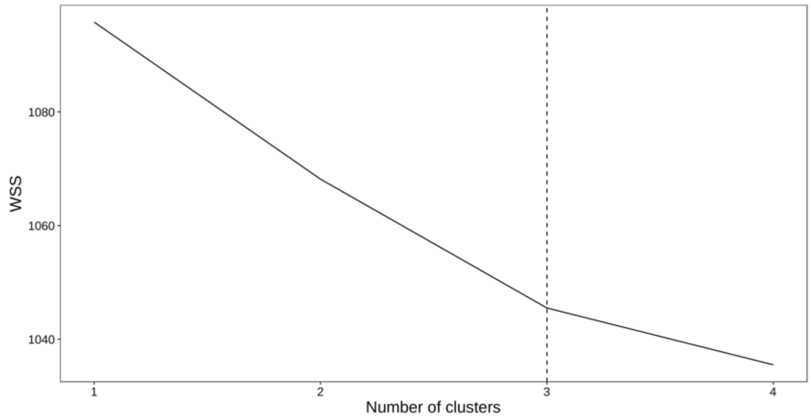

b

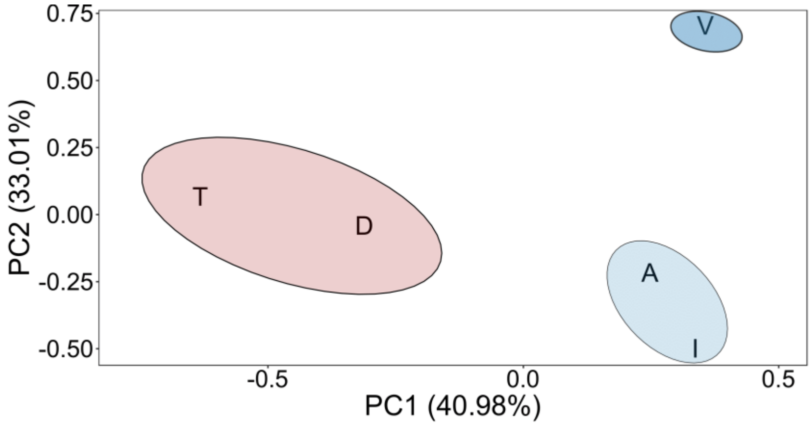

Figure S2. (**a**) The optimal number of clusters for the k-means clusterization of all DIGE spots using the elbow method. The dashed line represents the number selected. (**b**) The PCA and k-means clustering results obtained by utilizing all the protein spots found in the 2D-DIGE gel for strain 800/3 vegetative culture (V), strain 800/3 virulent sporulating (S) culture, and strain 800/3-15 avirulent sporulating (A).

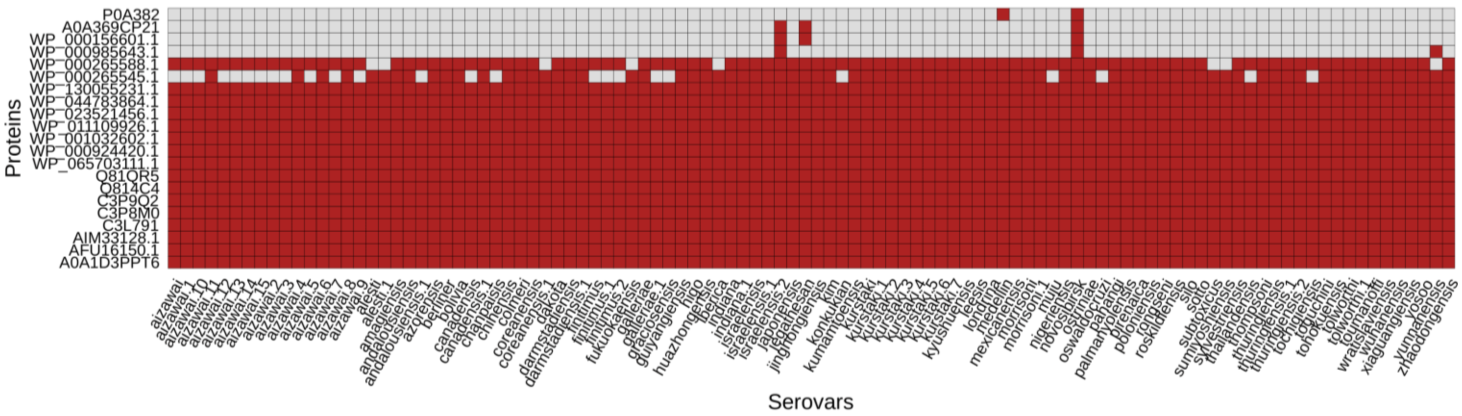

Figure S3: The presence/absence of proteins and the corresponding genes among genome assemblies with regard to their serovars' attribution. The red color denotes a gene's presence in a genome.

a

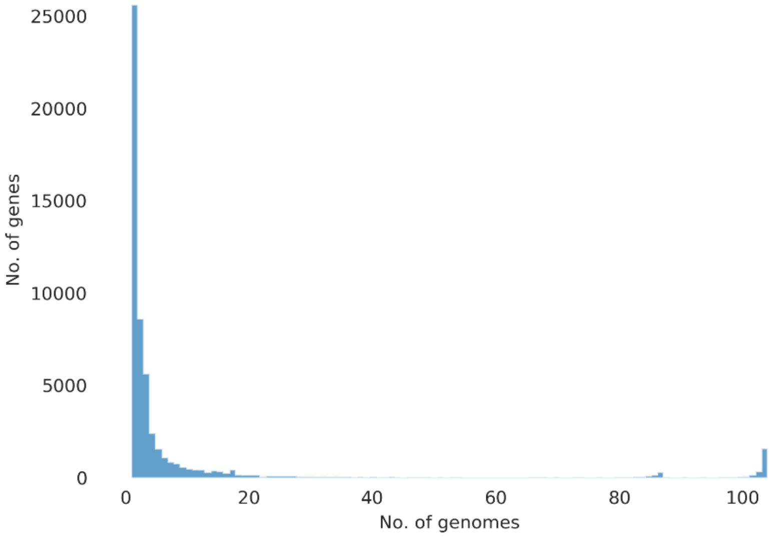

b

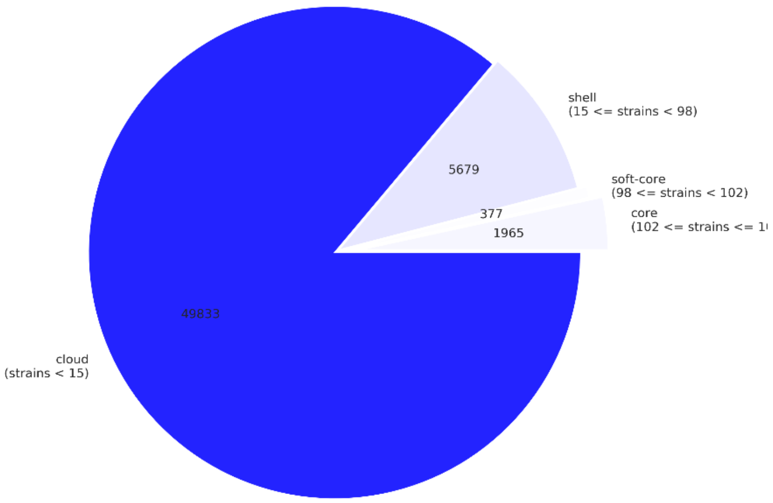

c

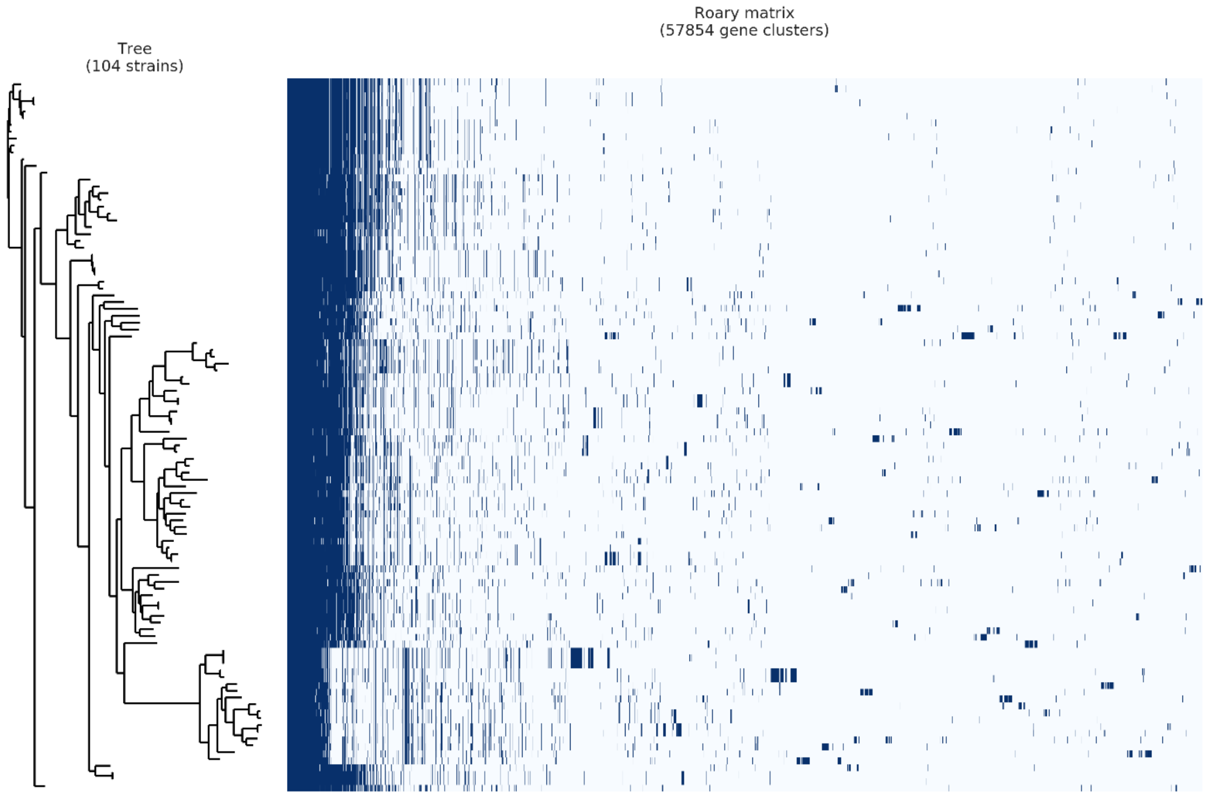

Figure S4: Visualization of Roary-obtained pangenome reconstructed on 104 pre-filtered Bt assemblies. (a) The link between the number of genomes and the number of new genes in the pangenome. (b) A pie chart visualizing the number of core and cloud genes in the pangenome. (c) The presence/absence matrix based on the accessory genes and the corresponding tree. The detailed description of the plots obtained is available at http://sanger-pathogens.github.io/Roary/.

a

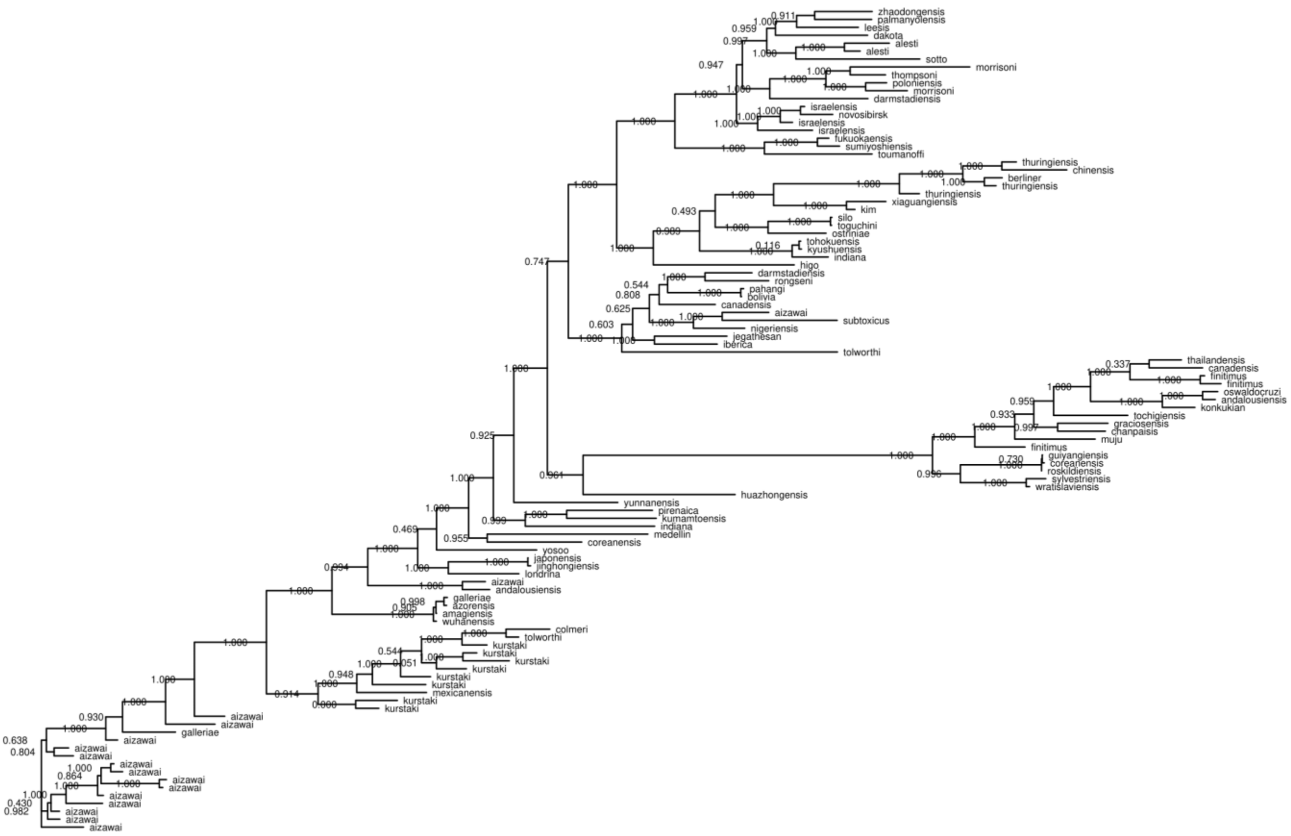
zhaodongensis
palmanyolensis
leesis
dakota
alesti
alesti
sotto
morrisoni
thompsoni
poloniensis
morrisoni
darmstadiensis
israelensis
novosibirsk
israelensis
israelensis
fukuokaensis
sumiyoshiensis
toumanoffi
thuringiensis
chinensis
berliner
thuringiensis
thuringiensis
xiaguangiensis
kim
silo
toguchini
ostriniae
tohokuensis
kyushuensis
indiana
higo
darmstadiensis
rongseni
pahangi
bolivia
canadensis
aizawai
subtoxicus
nigeriensis
jegathesan
iberica
tolworthi
thailandensis
canadensis
finitimus
finitimus
oswaldocruzi
andalousiensis
konkukian
tochigiensis
graciosensis
chanpaisis
muju
finitimus
guiyangiensis
coreanensis
roskildiensis
sylvestriensis
wratislaviensis
huazhongensis
yunnanensis
pirenaica
kumamtoensis
indiana
medellin
coreanensis
yosoo
japonensis
jinghongiensis
londrina
aizawai
andalousiensis
galleriae
azorensis
amagiensis
wuhanensis
colmeri
tolworthi
kurstaki
kurstaki
kurstaki
kurstaki
kurstaki
kurstaki
mexicanensis
kurstaki
kurstaki
aizawai
aizawai
galleriae
aizawai
aizawai
aizawai
aizawai
aizawai
aizawai
aizawai
aizawai
aizawai
aizawai
aizawai
aizawai

b

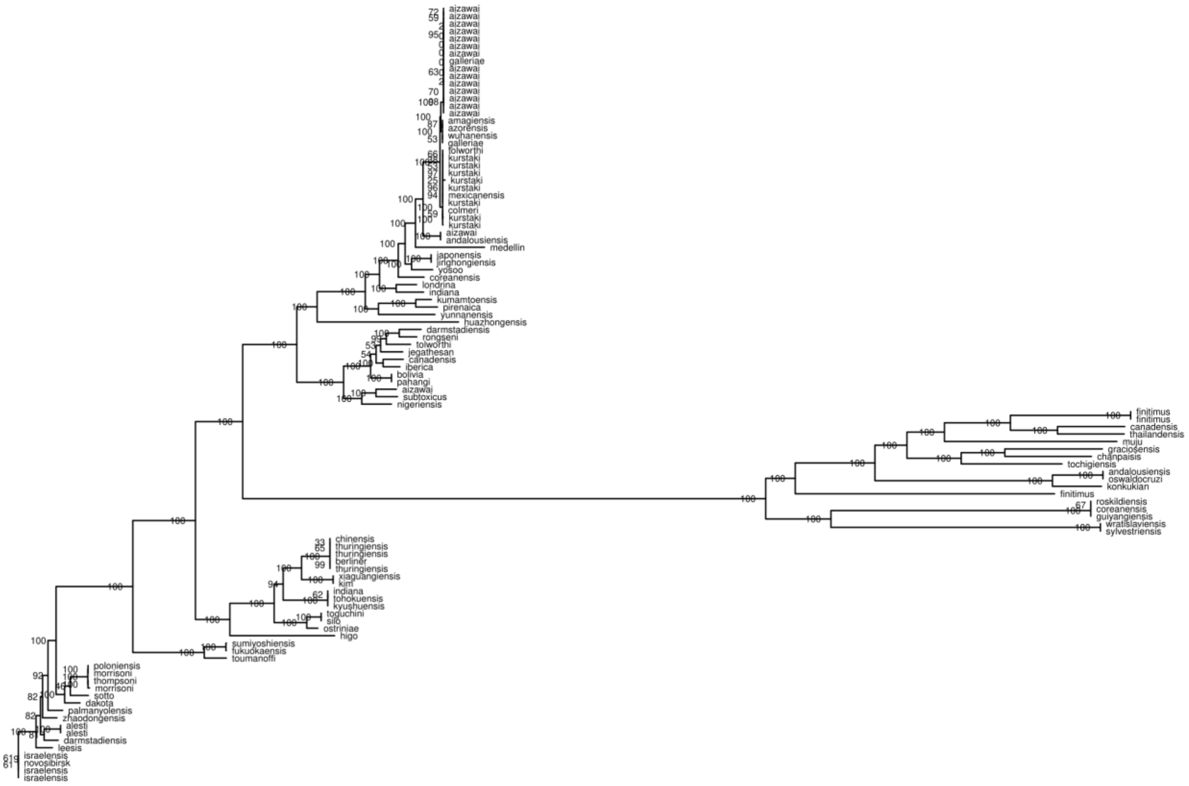
aizawai
galleriae
amagiensis
azorensis
wuhanensis
galleriae
tolworthi
kurstaki
mexicanensis
colmeri
andalousiensis
medellin
japonensis
jinghongiensis
yosoo
coreanensis
londrina
indiana
kumamtoensis
pirenaica
yunnanensis
huazhongensis
darmstadiensis
rongseni
tolworthi
jegathesan
canadensis
iberica
bolivia
pahangi
aizawai
subtoxicus
nigeriensis
finitimus
canadensis
thailandensis
muju
graciosensis
chanpaisis
tochigiensis
andalousiensis
oswaldocruzi
konkukian
roskildiensis
coreanensis
guiyangiensis
wratislaviensis
sylvestriensis
chinensis
thuringiensis
berliner
xiaguangiensis
kim
tohokuensis
kyushuensis
toguchini
silo
ostriniae
higo
sumiyoshiensis
fukuokaensis
toumanoffi
poloniensis
morrisoni
thompsoni
sotto
dakota
palmanyolensis
zhaodongensis
alesti
darmstadiensis
leesis
israelensis
novosibirsk

c

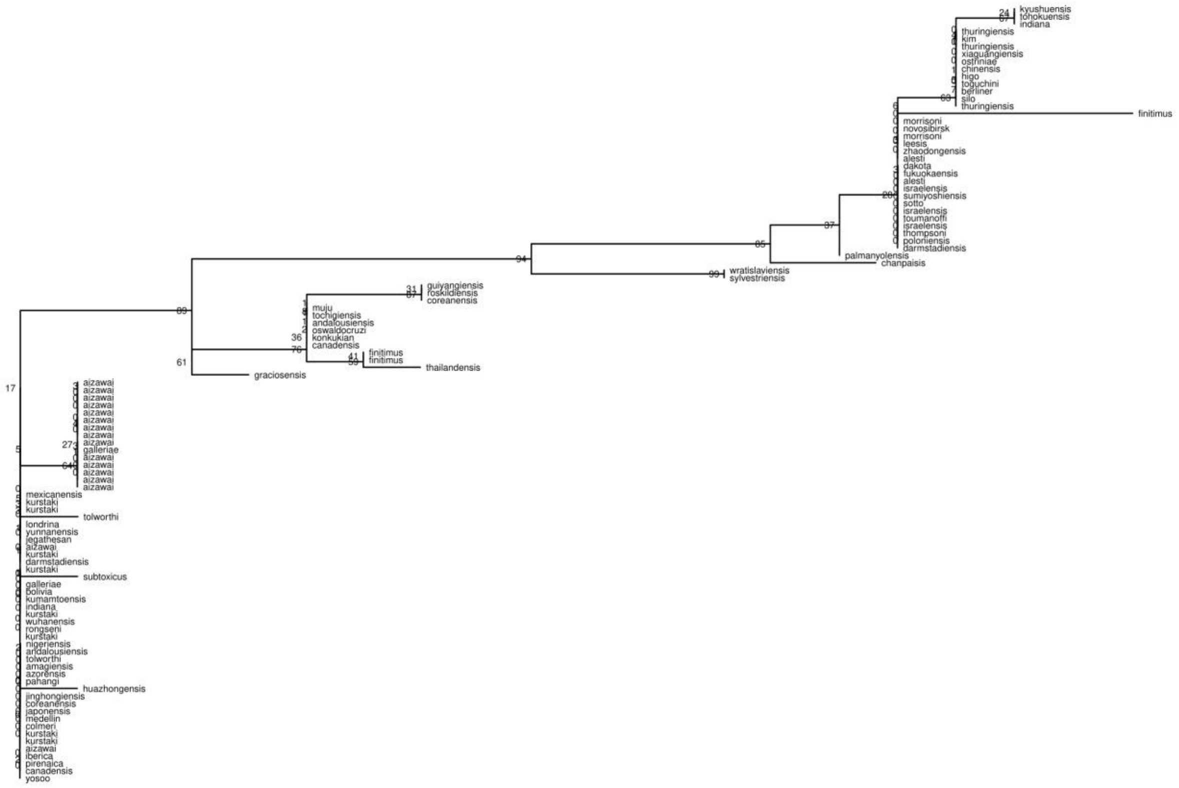

kyushuensis
tohokuensis
indiana
thuringiensis
kim
thuringiensis
xiaguangiensis
ostriniae
chinensis
higo
toguchini
berliner
silo
thuringiensis
finitimus
morrisoni
novosibirsk
morrisoni
leesis
zhaodongensis
alesti
dakota
fukuokaensis
alesti
israelensis
sumiyoshiensis
sotto
israelensis
toumanoffi
israelensis
thompsoni
poloniensis
darmstadiensis
palmanyolensis
chanpaisis
wratislaviensis
sylvestriensis
guiyangiensis
roskildiensis
coreanensis
muju
tochigiensis
andalousiensis
oswaldocruzi
konkukian
canadensis
finitimus
finitimus
thailandensis
graciosensis
aizawai
galleriae
mexicanensis
kurstaki
tolworthi
londrina
yunnanensis
jegathesan
darmstadiensis
subtoxicus
bolivia
kumamtoensis
wuhanensis
rongseni
nigeriensis
amagiensis
azorensis
pahangi
huazhongensis
jinghongiensis
japonensis
medellin
colmeri
iberica
pirenaica
yosoo

d

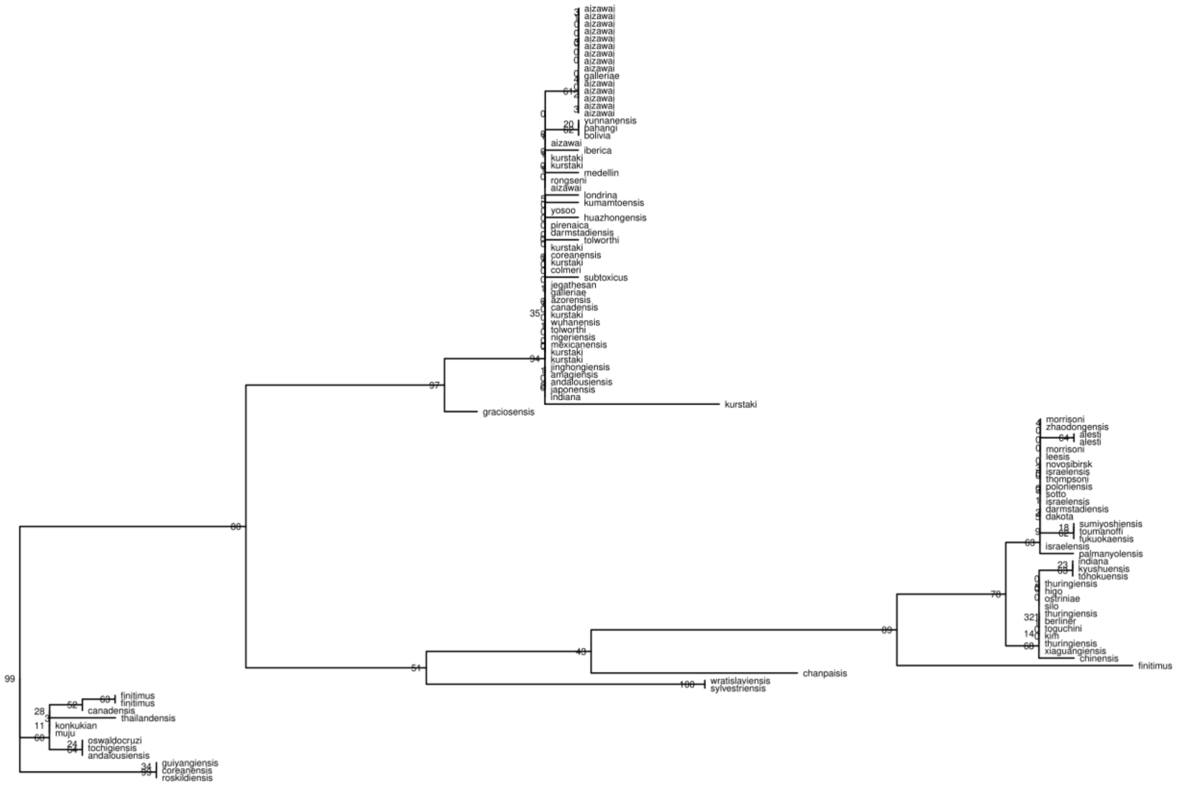

aizawai
galleriae
yunnanensis
pahangi
bolivia
iberica
kurstaki
medellin
rongseni
londrina
kumamtoensis
yosoo
huazhongensis
pirenaica
darmstadiensis
tolworthi
coreanensis
colmeri
subtoxicus
jegathesan
azorensis
canadensis
wuhanensis
nigeriensis
mexicanensis
jinghongiensis
amagiensis
andalousiensis
japonensis
indiana
graciosensis
morrisoni
zhaodongensis
alesti
leesis
novosibirsk
israelensis
thompsoni
poloniensis
sotto
dakota
sumiyoshiensis
toumanoffi
fukuokaensis
palmanyolensis
kyushuensis
tohokuensis
thuringiensis
higo
ostriniae
silo
berliner
toguchini
kim
xiaguangiensis
chinensis
finitimus
chanpaisis
wratislaviensis
sylvestriensis
canadensis
thailandensis
konkukian
muju
oswaldocruzi
tochigiensis
guiyangiensis
coreanensis
roskildiensis

e

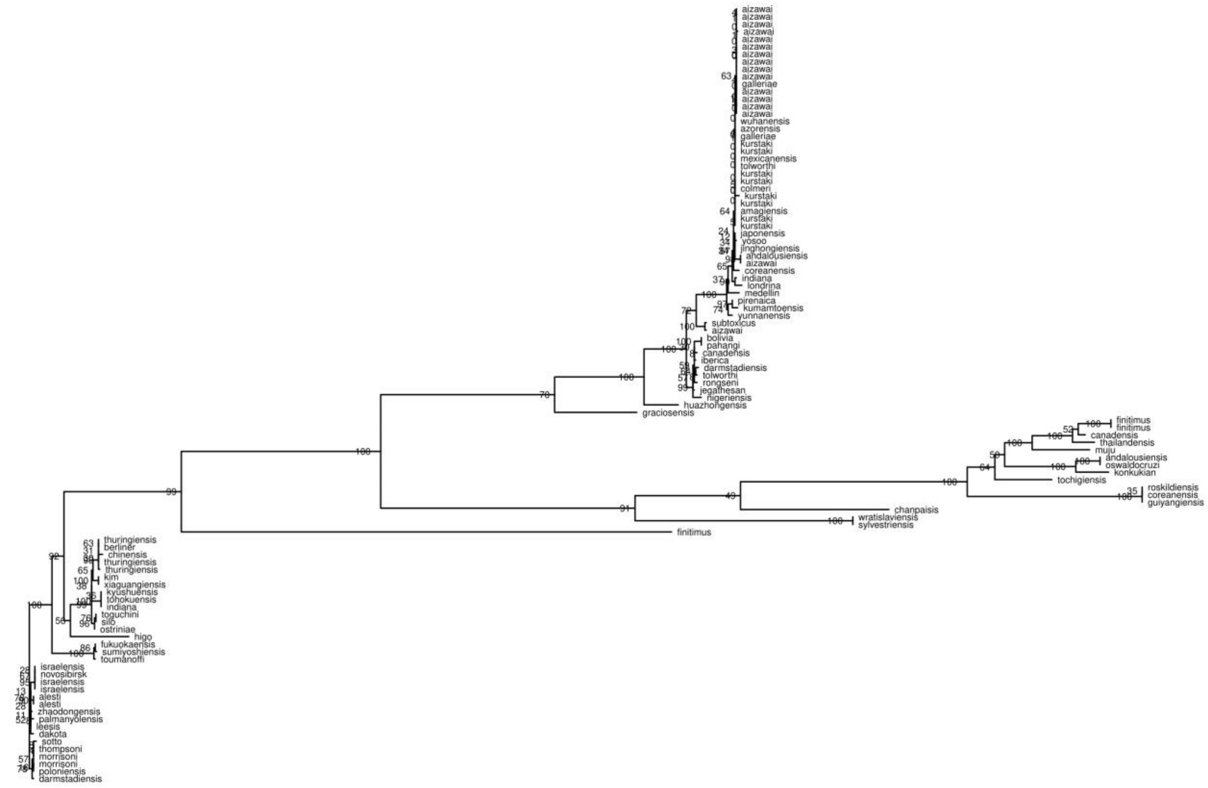

f

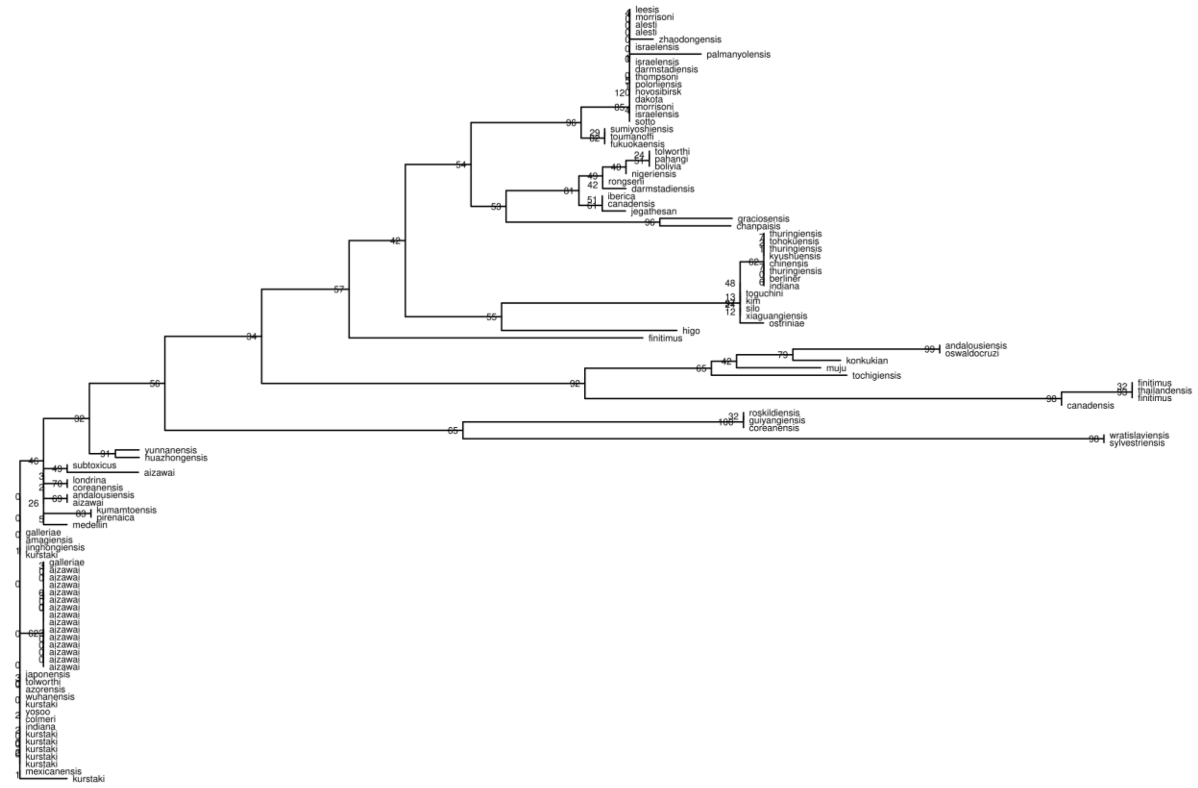

g

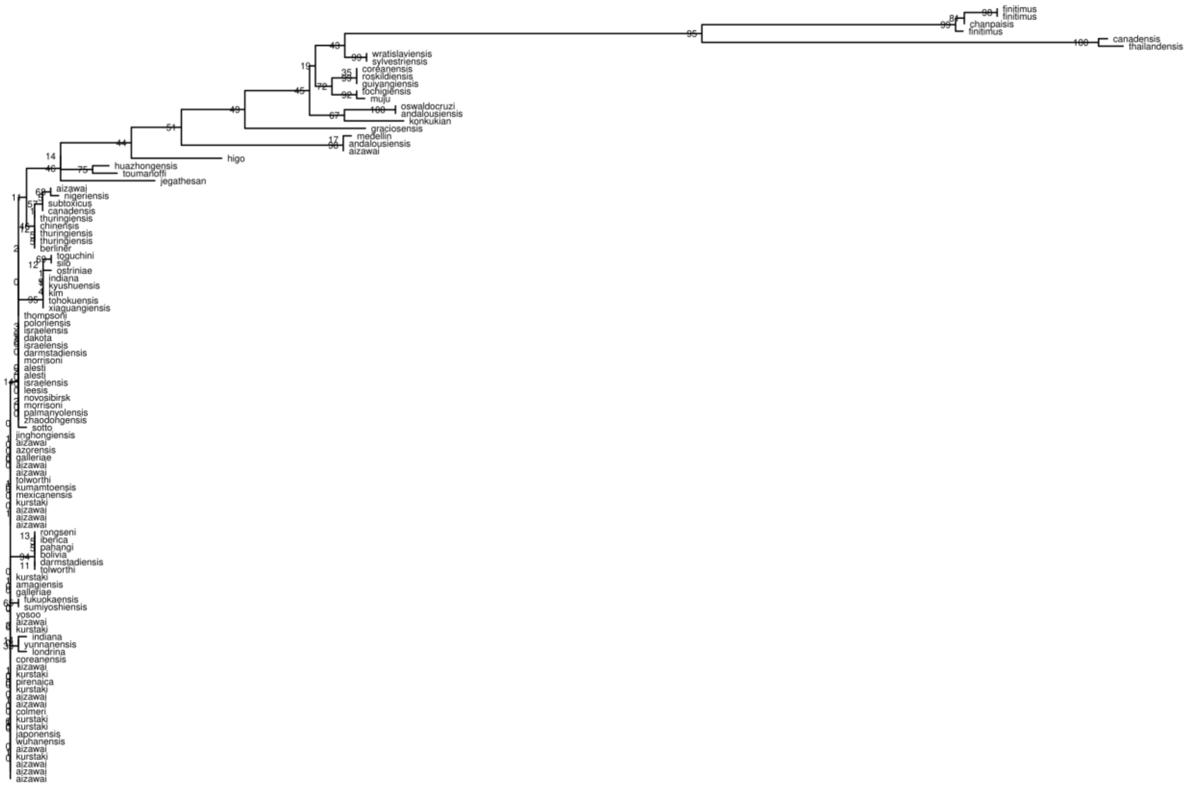

h

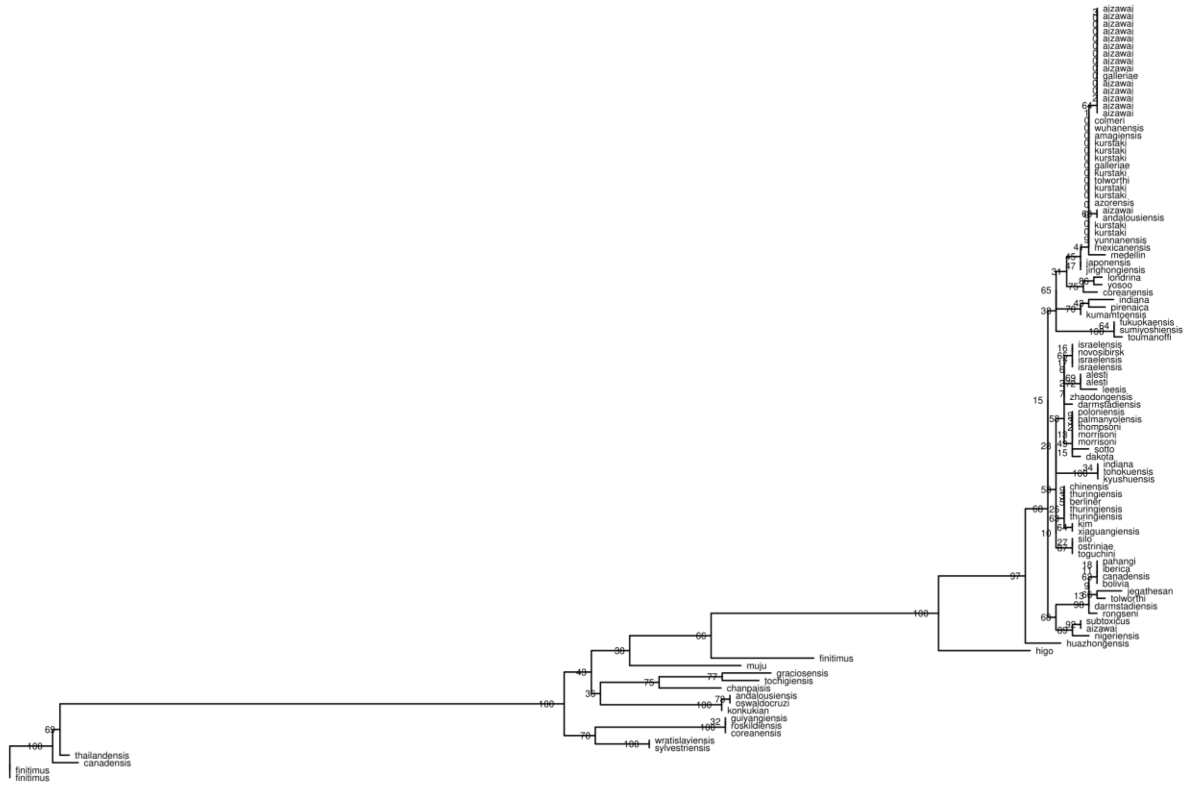

i

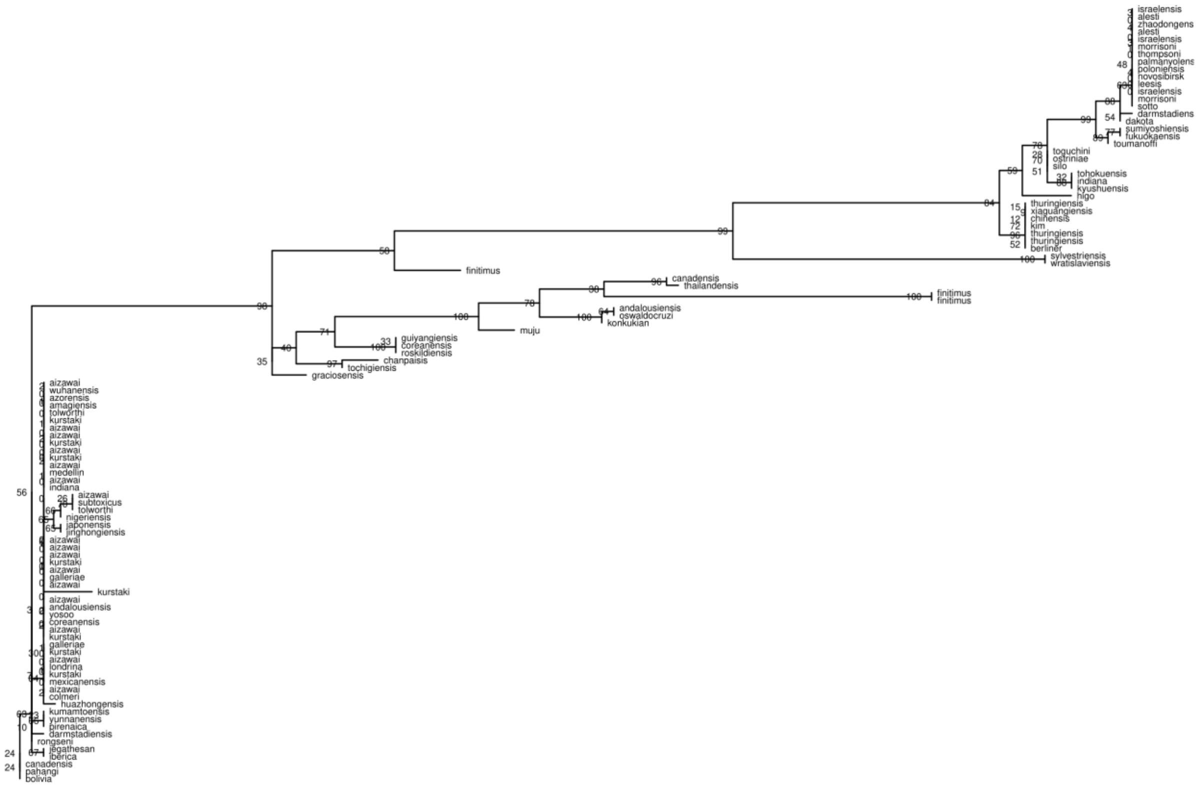

j

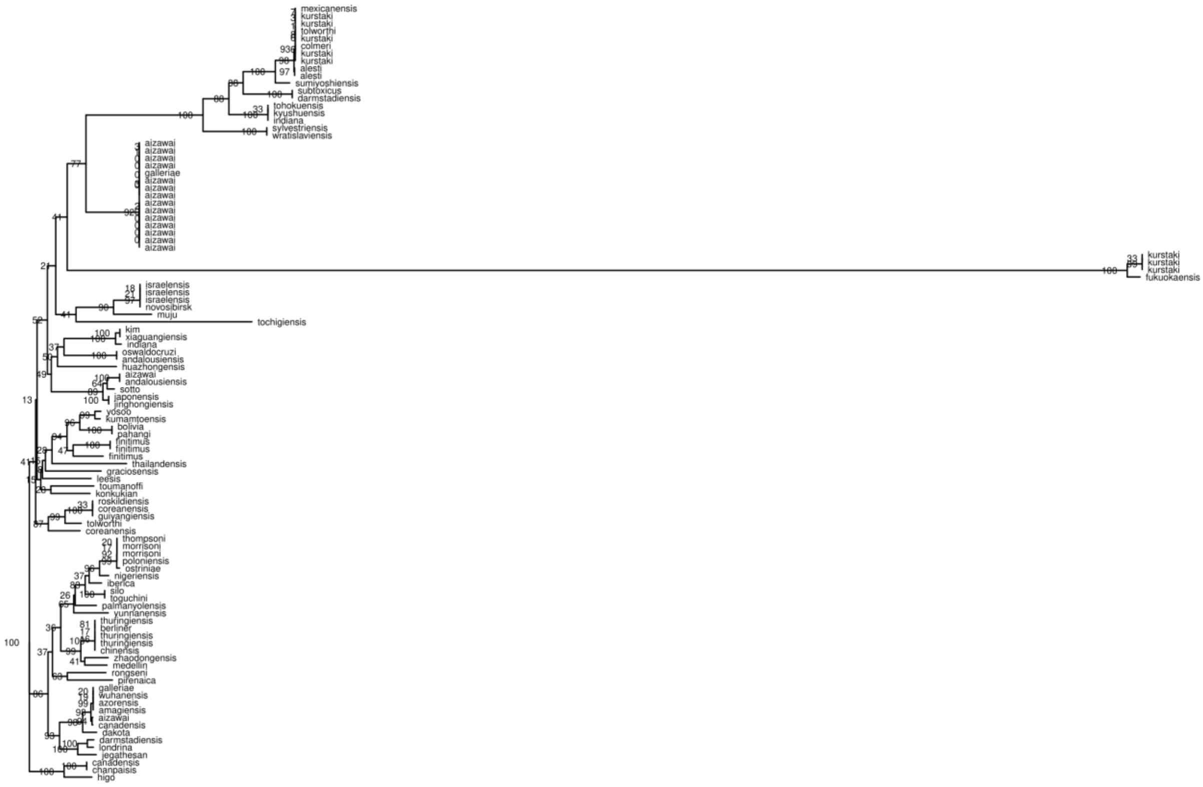

k

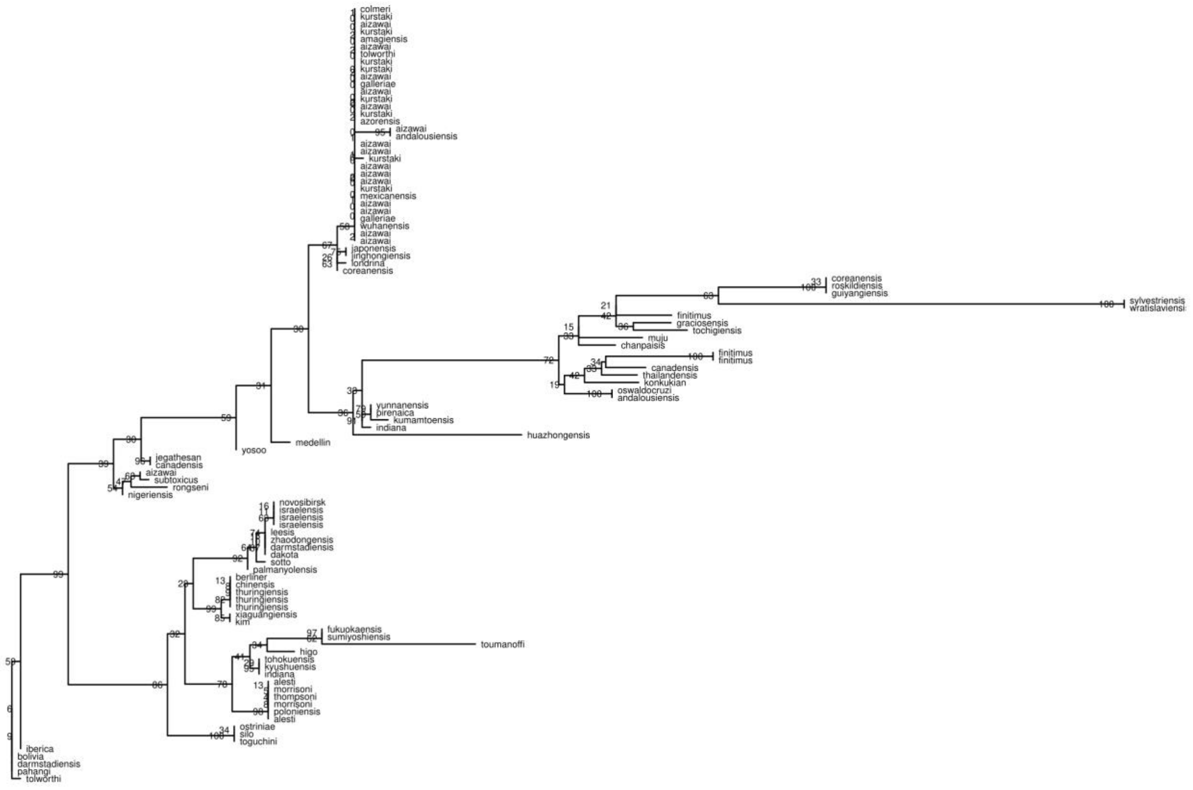

l

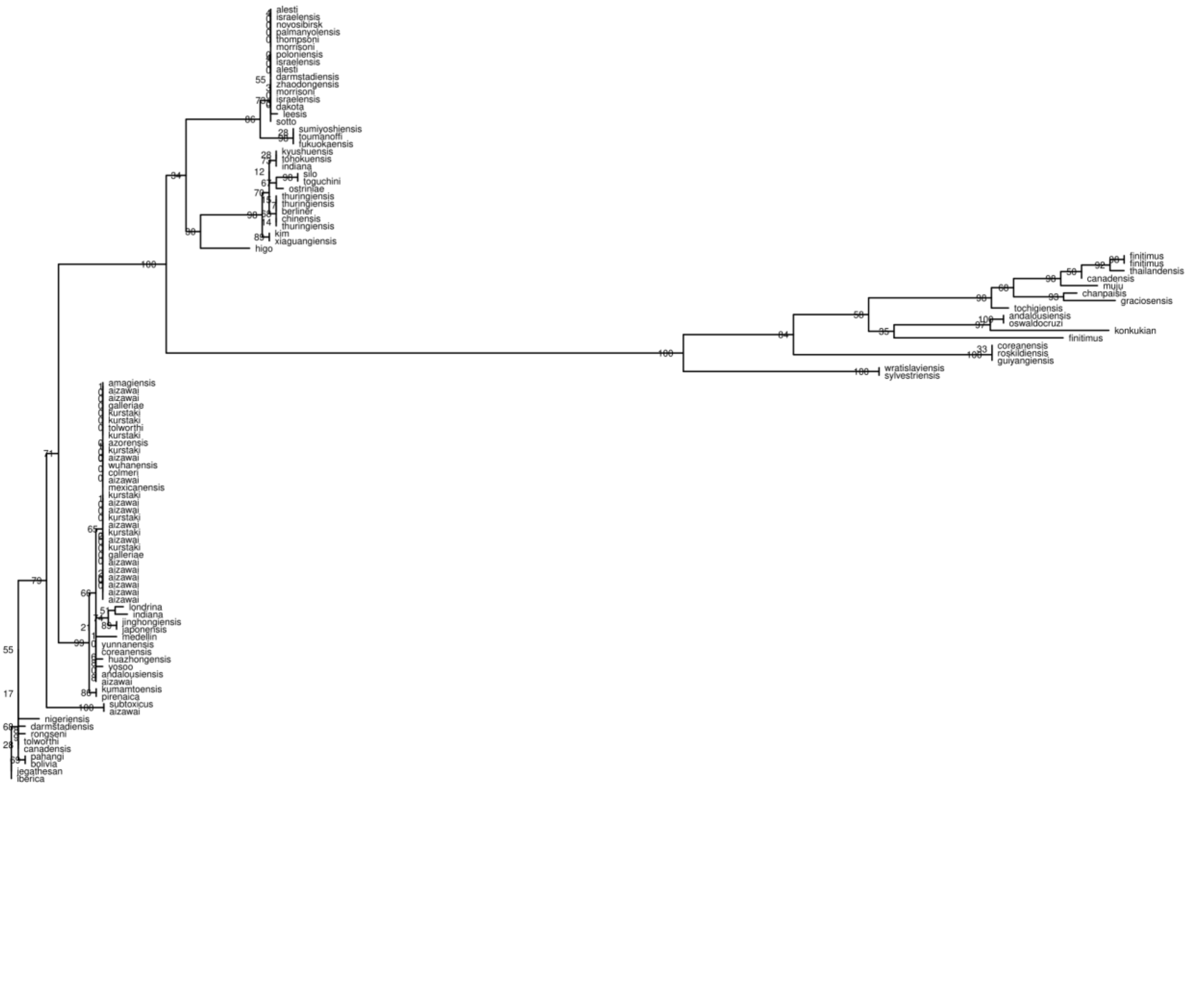

m

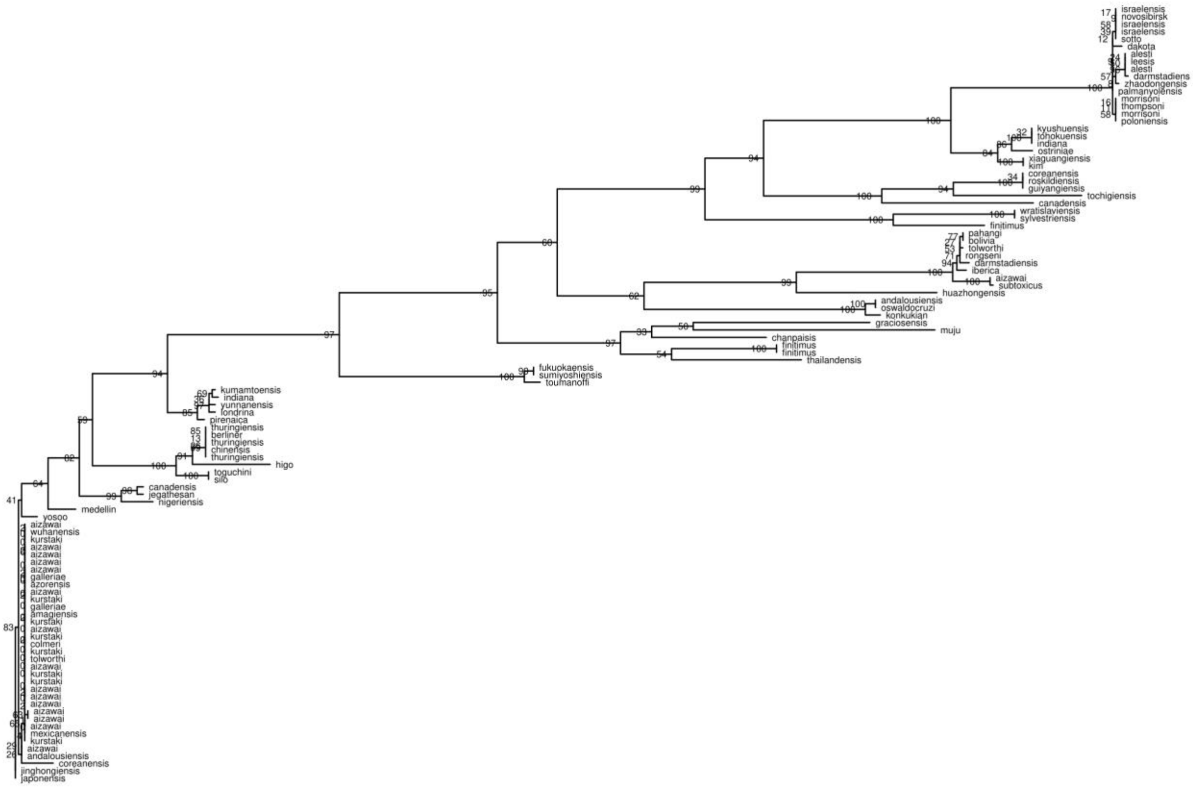

n

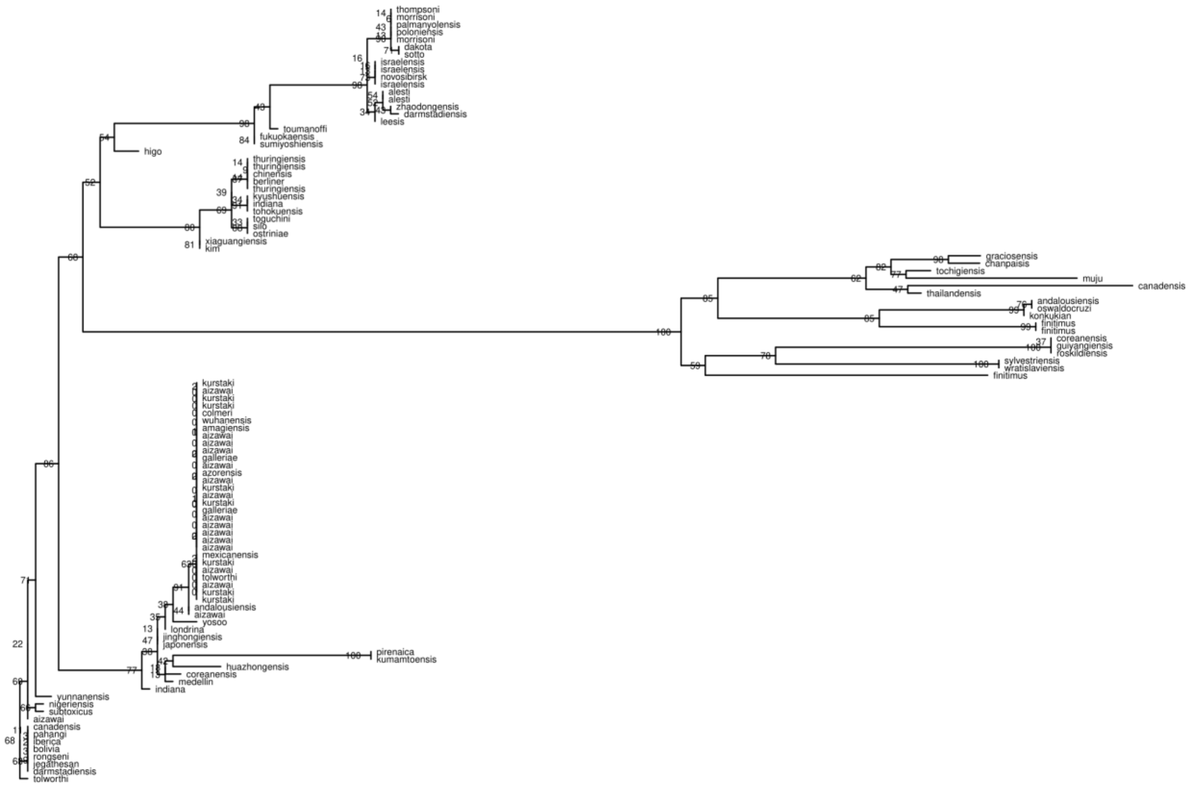

o

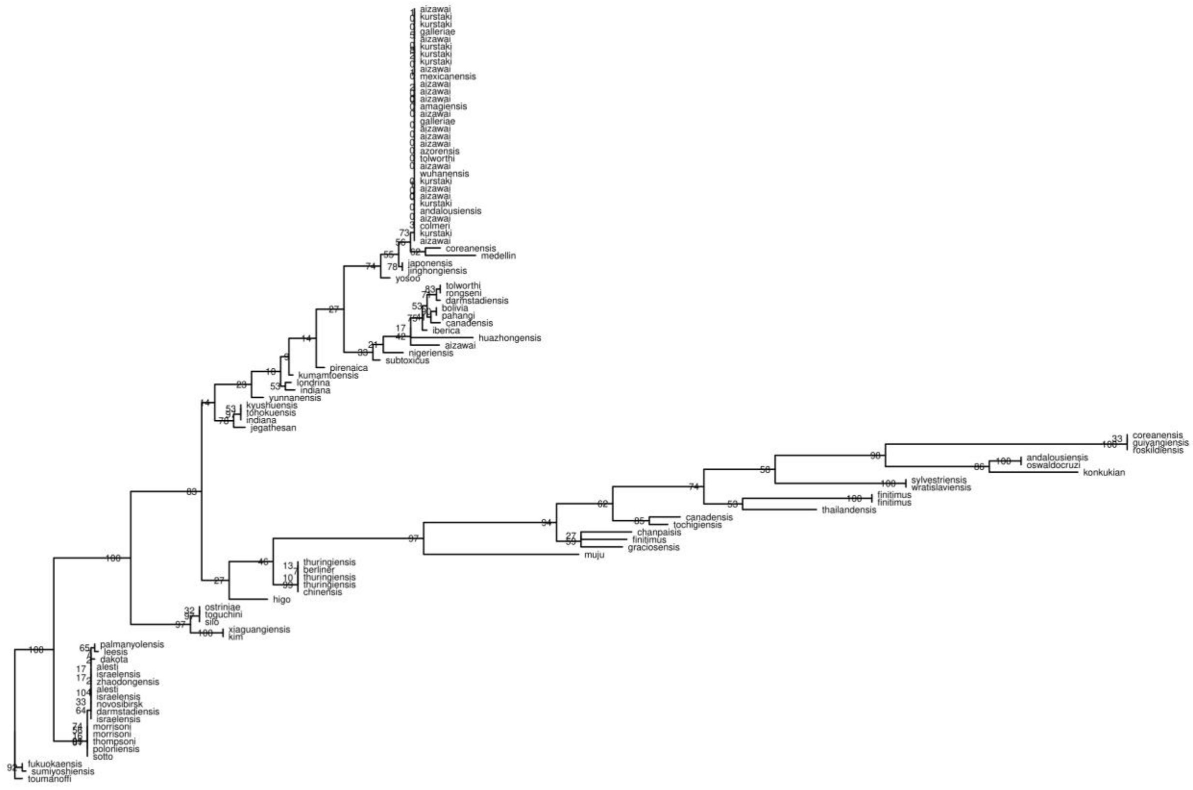

p

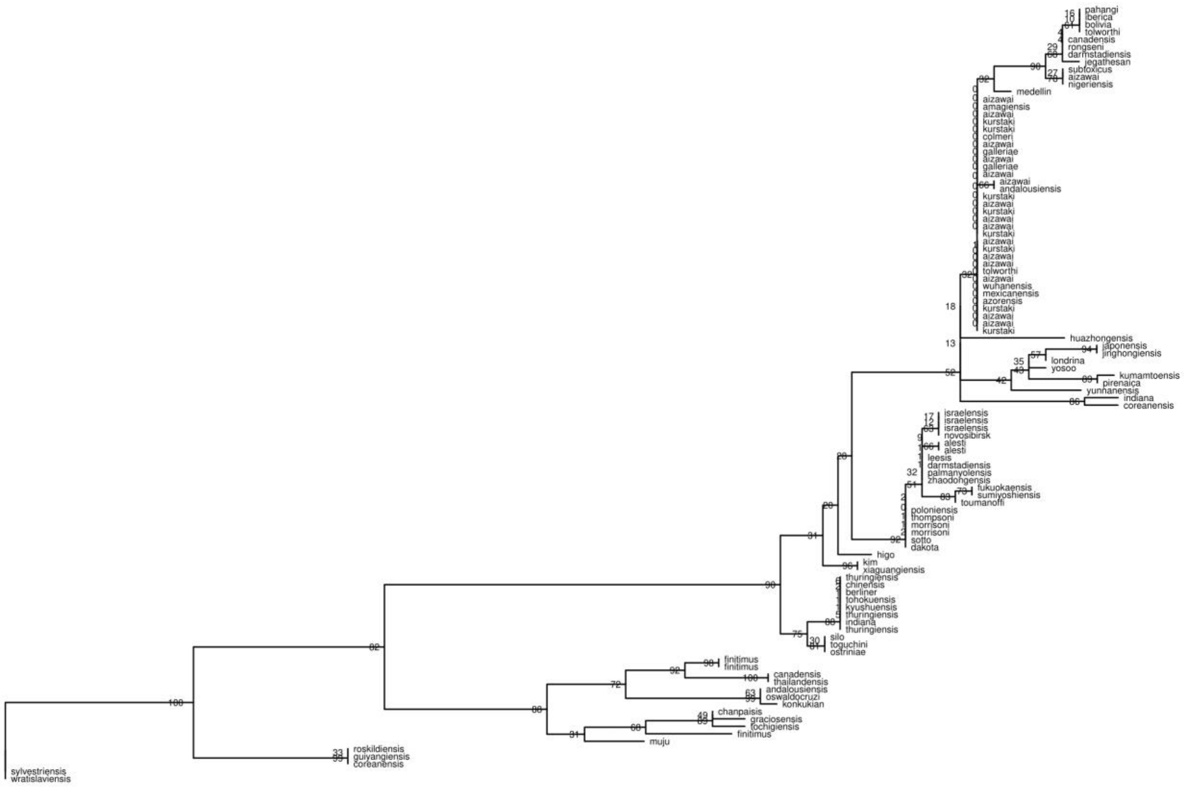

q

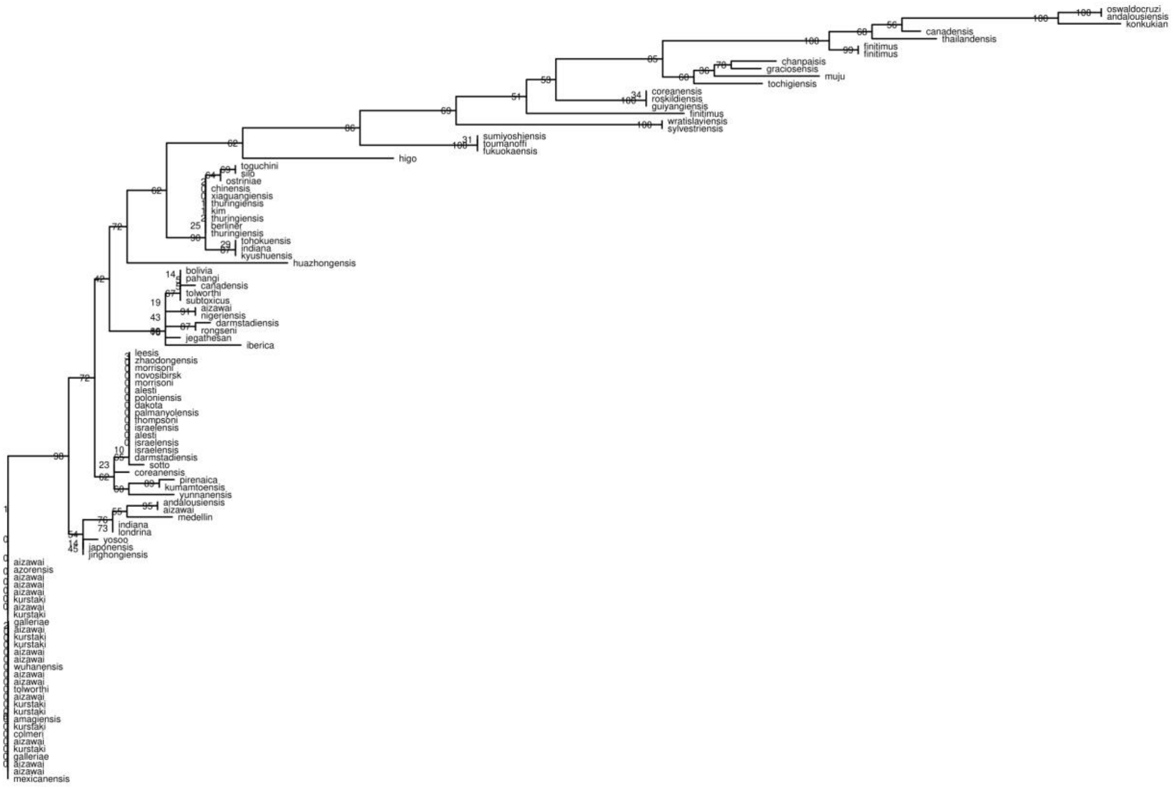

r

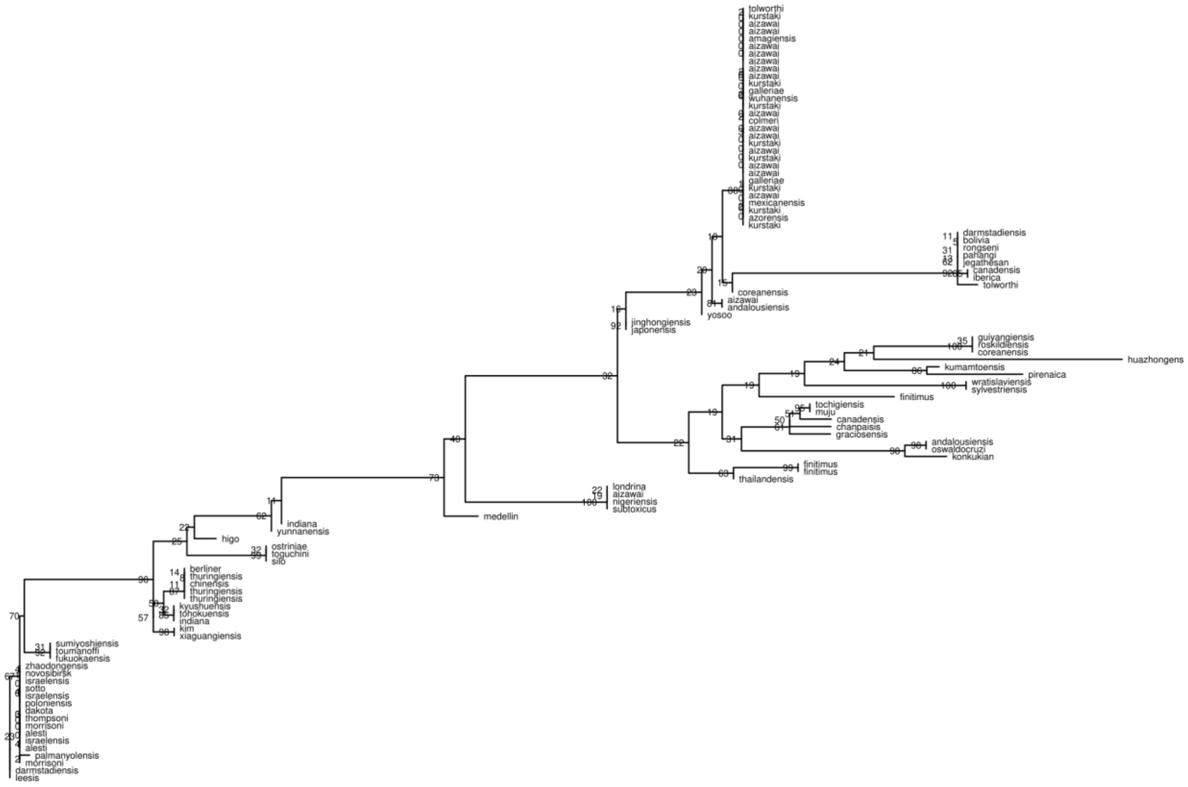

s

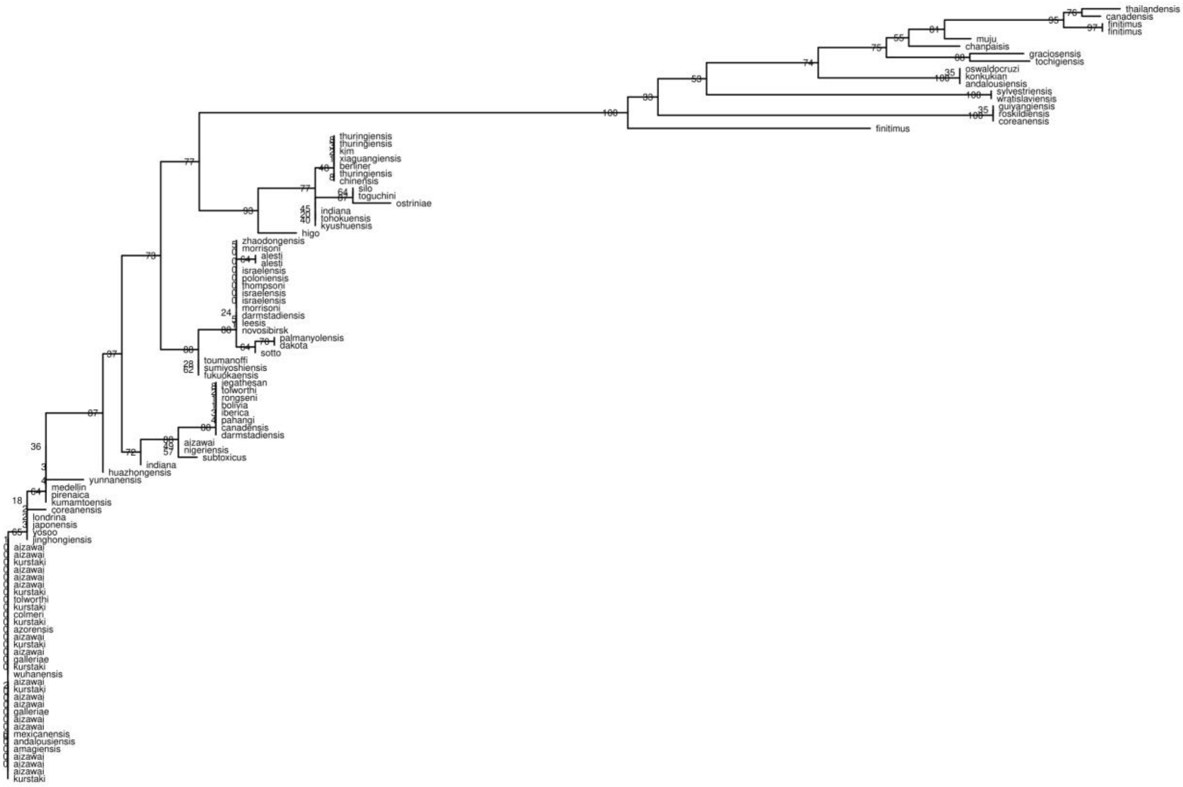
thailandensis
canadensis
finitimus
finitimus
muju
chanpaisis
graciosensis
tochigiensis
oswaldocruzi
konkukian
andalousiensis
sylvestriensis
wratislaviensis
guiyangiensis
roskildiensis
coreanensis
finitimus
thuringiensis
thuringiensis
kim
xiaguangiensis
berliner
thuringiensis
chinensis
silo
toguchini
ostriniae
indiana
tohokuensis
kyushuensis
higo
zhaodongensis
morrisoni
alesti
alesti
israelensis
poloniensis
thompsoni
israelensis
israelensis
morrisoni
darmstadiensis
leesis
novosibirsk
palmanyolensis
dakota
sotto
toumanoffi
sumiyoshiensis
fukuokaensis
jegathesan
tolworthi
rongseni
bolivia
iberica
pahangi
canadensis
darmstadiensis
aizawai
nigeriensis
subtoxicus
indiana
huazhongensis
yunnanensis
medellin
pirenaica
kumamtoensis
coreanensis
londrina
japonensis
yosoo
jinghongiensis
aizawai
aizawai
kurstaki
aizawai
aizawai
aizawai
kurstaki
tolworthi
kurstaki
colmeri
kurstaki
azorensis
aizawai
kurstaki
aizawai
galleriae
kurstaki
wuhanensis
aizawai
kurstaki
aizawai
aizawai
galleriae
aizawai
aizawai
mexicanensis
andalousiensis
amagiensis
aizawai
aizawai
aizawai
kurstaki

t

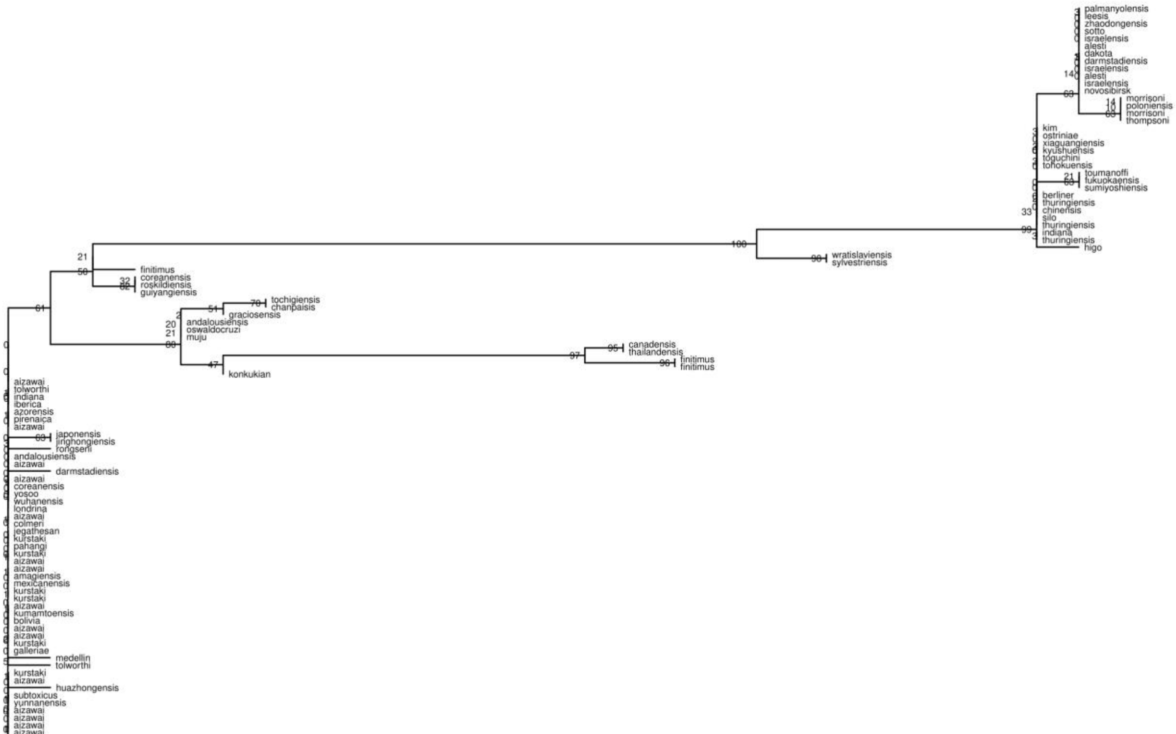
palmanyolensis
leesis
zhaodongensis
sotto
israelensis
alesti
dakota
darmstadiensis
israelensis
alesti
israelensis
novosibirsk
morrisoni
poloniensis
morrisoni
thompsoni
kim
ostriniae
xiaguangiensis
kyushuensis
toguchini
tohokuensis
toumanoffi
fukuokaensis
sumiyoshiensis
berliner
thuringiensis
chinensis
silo
thuringiensis
indiana
thuringiensis
higo
wratislaviensis
sylvestriensis
finitimus
coreanensis
roskildiensis
guiyangiensis
tochigiensis
chanpaisis
graciosensis
andalousiensis
oswaldocruzi
muju
canadensis
thailandensis
finitimus
finitimus
konkukian
aizawai
tolworthi
indiana
iberica
azorensis
pirenaica
aizawai
japonensis
jinghongiensis
rongseni
andalousiensis
aizawai
darmstadiensis
aizawai
coreanensis
yosoo
wuhanensis
londrina
aizawai
colmeri
jegathesan
kurstaki
pahangi
kurstaki
aizawai
aizawai
amagiensis
mexicanensis
kurstaki
kurstaki
aizawai
kumamtoensis
bolivia
aizawai
aizawai
kurstaki
galleriae
medellin
tolworthi
kurstaki
aizawai
huazhongensis
subtoxicus
yunnanensis
aizawai
aizawai
aizawai
aizawai
kurstaki
nigeriensis
kurstaki
canadensis
aizawai
galleriae

u

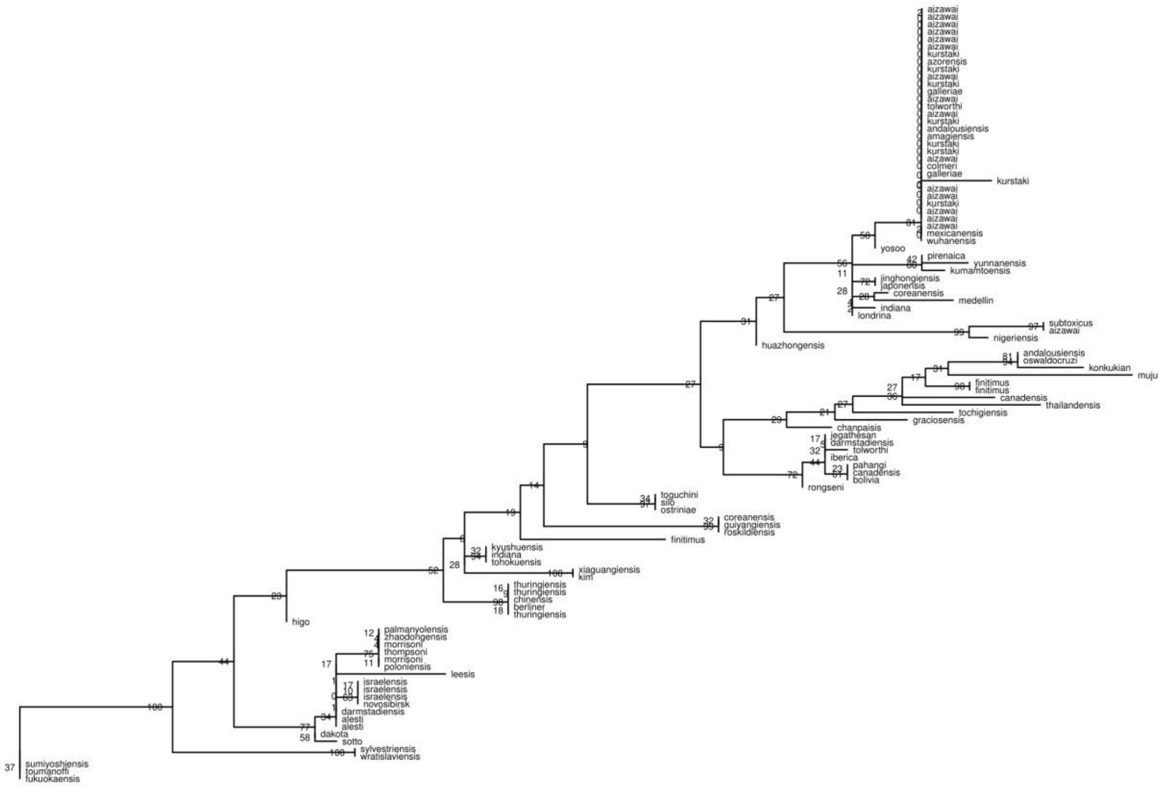

Figure S5: All phylogenomic and single-loci phylogenetic trees based on Bt-genomes. Trees ere reconstructed using raxml-ng. Supporting values for 1000 bootstrap replicates are shown. Used evolutionary models and mean supporting values are presented in Table S8. **(a)** Presence/absence-based tree. (**b**) Core SNPs' tree. (**c**) Protein *gyrB*. (**d**) Concatenated protein *gyrB* and *gyrA* alignments-based tree.(**e**) Concatenated nucleotide *gyrB* and *gyrA* alignments-based tree (**f**) flagellin orthologs. (**g**) *calY*. (**h**) *dnaK*. (**i**) *fusA*. (**j**) *atpD*. (**k)** *groEL*. (**l**) *guaB*. (**m**) *inhA*. (**n**) *mmsA*. (**o**) *nprB*. (**p**) *phbB*. (**q**) *rocA*. (**r**) *rph*. (**s**) *sucC*. (**t**) *tuf*. (**u**) *yjlD*.

a

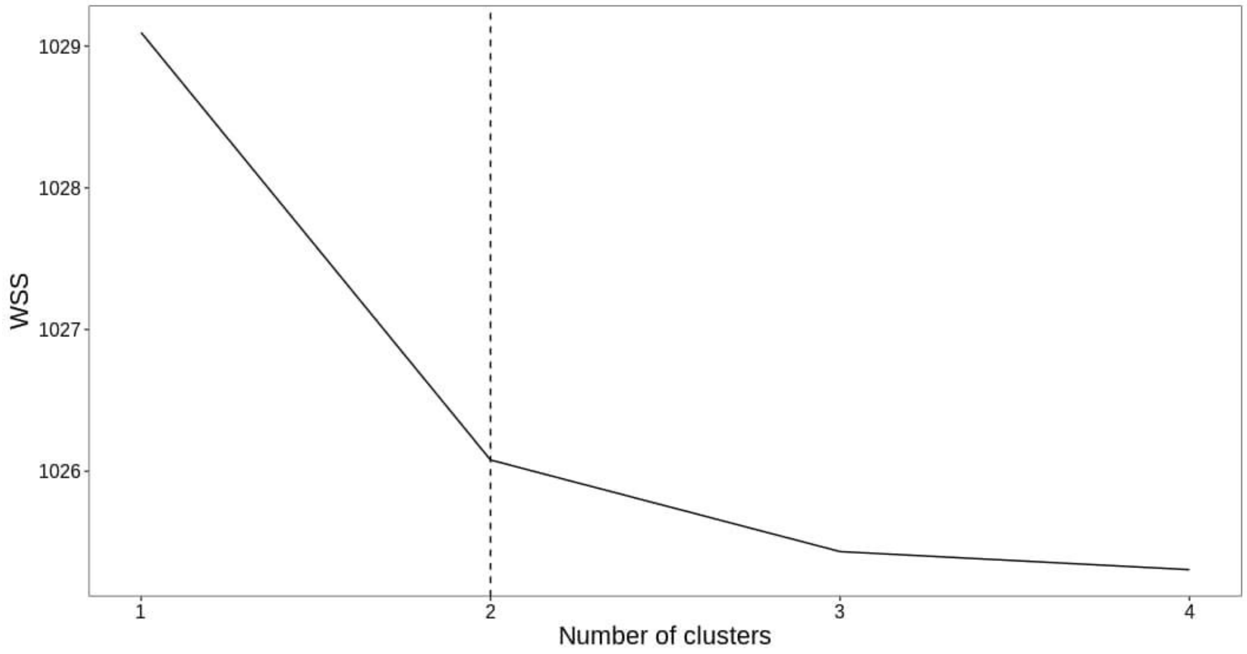

b

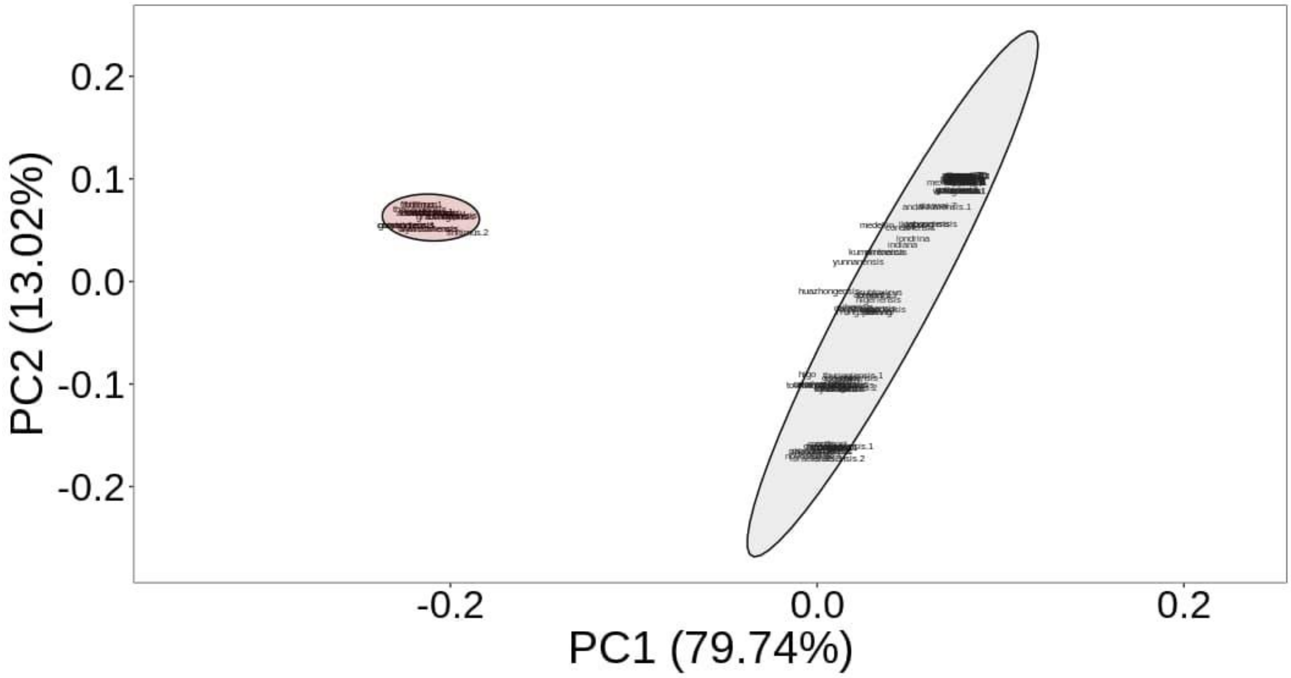

Figure S6: (**a**) The optimal number of clusters for the k-means clusterization of the data based on the ANI matrix. (**b**) The results of k-means clusterization of the serovars.

a

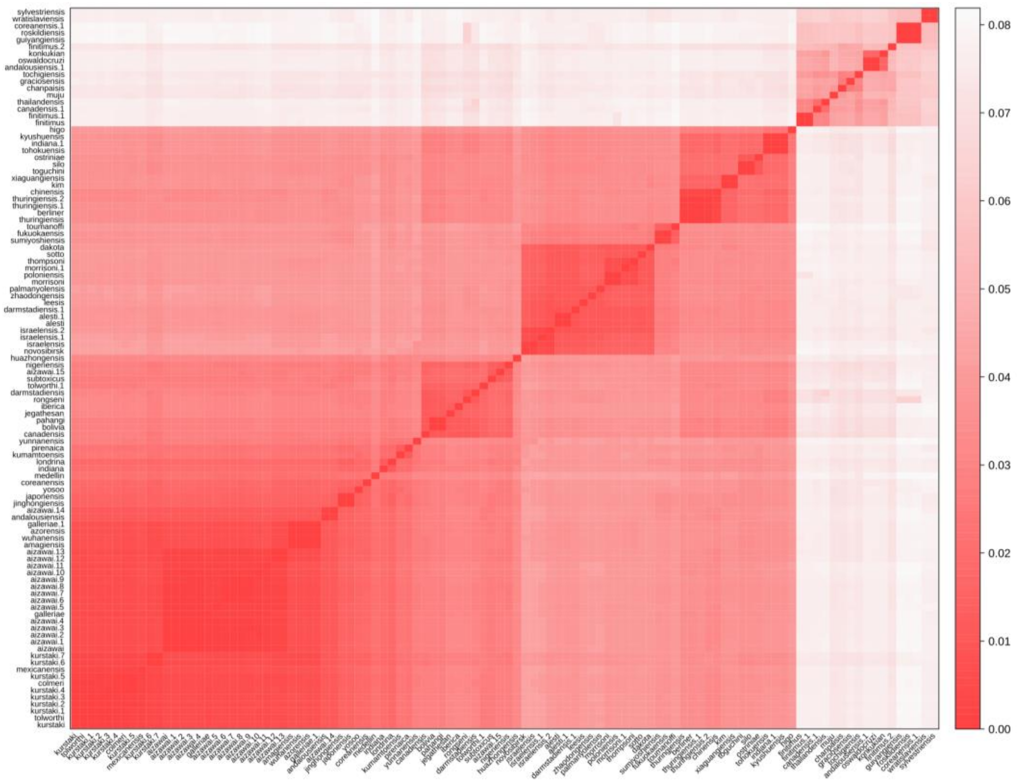

b

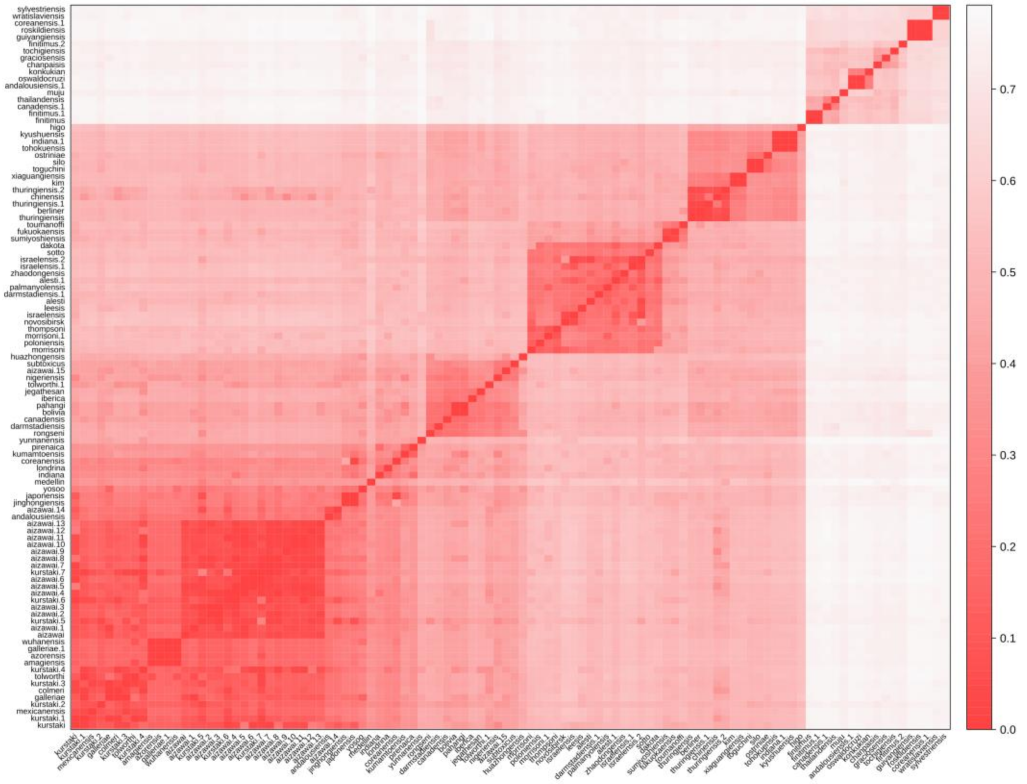

Figure S7: Heatmap-visualisation of clusters based on mash-distance and mean genome identity. (**a**) A mash distance-based heatmap. The intensity of the color is proportional to ANI (1-mash distance). (**b**) A minimap-2-based heatmap. The intensity of the color is proportional to the mean identity between genomes.

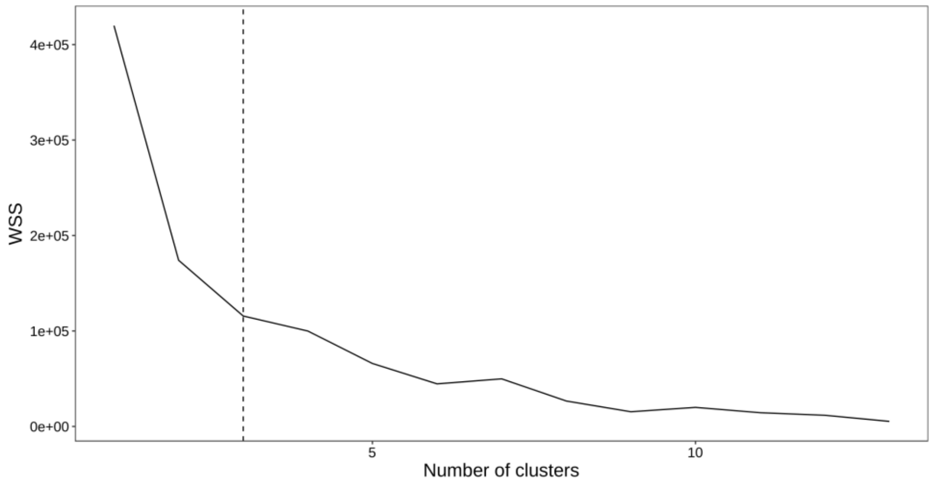

Figure S8: The optimal number of clusters for the k-means clusterization of the data based on subtrees' length containing all representatives of each serovar using the elbow method. The dashed line represents the number selected (3) depicted in Figure 5d of the main text.

.
